# Healthcare experiences of refugees and asylum-seekers resettled in non-metropolitan high-income settings: a systematic review and thematic synthesis of qualitative evidence

**DOI:** 10.1186/s12939-026-02933-9

**Published:** 2026-07-14

**Authors:** K. J. Woodward, R. B. Kirk, G. A. Borchert, N. Roberts, C. Pope, S. Tierney

**Affiliations:** 1 Nuffield Department of Primary Care Health Sciences, University of Oxford, Oxford, UK; 2 School of Medicine and Dentistry, Griffith University, Gold Coast, Australia; 3 Division of Cardiovascular Medicine, Radcliffe Department of Medicine, University of Oxford, Oxford, UK; 4 Nuffield Laboratory of Ophthalmology, Nuffield Department of Clinical Neurosciences, University of Oxford, Oxford, UK; 5 Bodleian Health Care Libraries, University of Oxford, Oxford, UK

**Keywords:** Refugee health, Asylum-seekers, Rural health, Health equity, Healthcare experiences, Qualitative synthesis

## Abstract

**Background:**

To address imbalances in the distribution of refugees and asylum-seekers during resettlement in high-income countries, some governments are employing dispersal strategies to extend resettlement beyond major cities. However, with populations in non-metropolitan areas facing well-documented differences in healthcare access and outcomes compared to their city counterparts, it is essential to consider the human impact of these resettlement policies. The primary aim of this study was to understand the healthcare experiences of refugees and asylum-seekers living in rural, regional, and other non-metropolitan areas of high-income countries. The secondary aim was to identify factors that influence these experiences.

**Methods:**

A thematic synthesis of qualitative or mixed-methods studies was undertaken. We searched seven electronic databases, Medline(OvidSP), Embase(OvidSP), PsycINFO(OvidSP), CINAHL(EBSCOHost), Social Science Citation Index(Web of Science Core Collection), BIOSIS Citation Index(Web of Science) and ASSIA(Proquest), up to October 6, 2025, for articles in English reporting qualitative data on the healthcare experiences of refugees and asylum seekers. At least two reviewers contributed to all review stages, including quality assessment.

**Results:**

Seventeen articles representing fourteen studies were included, capturing the experiences of 347 refugees and asylum-seekers. We developed descriptive themes characterising everyday healthcare experiences, from which analytical themes were produced to describe underlying determinants of these experiences: *The Domains of Influence, The Power of the Social, and The Rural Effect*. These findings explain how the convergence of a refugee or asylum-seeker’s individual, sociocultural, environmental, and political realities shape their healthcare experiences after resettlement in a rural area. They highlight how rurality and place interact with social and structural determinants of health to intensify both the supportive dimensions of small-community life and the barriers and inequities produced by limited resources, distance, and geographic isolation.

**Conclusions:**

Despite policies promoting non-metropolitan resettlement in high-income countries, research on the healthcare experiences of refugees and asylum seekers in these areas remains limited. This review both characterises these experiences and suggests factors that shape them, which can inform healthcare providers and policymakers in addressing health inequities.

**Registration:**

PROSPERO CRD42024553190.

**Supplementary Information:**

The online version contains supplementary material available at https://doi.org/10.1186/s12939-026-02933-9.

## Introduction

The steadily rising number of forcibly displaced people means that refugee resettlement is a growing challenge for the international community. By mid-2025, there were 38.9 million refugees and asylum-seekers worldwide, representing a doubling of refugee numbers since 2015 [1].

Low- and middle-income countries (LMICs) host 71% of the world’s refugees. The large share of refugees in LMICs occurs largely due to geography, with 66% of people fleeing conflict or persecution to a neighbouring country. Low-income countries in particular host a disproportionately large share of refugees relative to both their population size and wealth [2]. Nevertheless, the United Nations General Assembly’s adoption of the Global Compact on Refugees demonstrates a growing global ambition to better share responsibility for refugee situations [3], and subsequent pressure on high-income countries (HICs) to improve access and support for resettlement [4].

Resettlement in a third country involves moving refugees from the country where they are seeking asylum to a long-term place of safety. Pathways to safe third countries, however, are often heavily regulated and influenced by government responses to global conflicts and political pressures. Despite consensus on the need to expand pathways [3], a large gap remains between the 2.9 million refugees requiring resettlement and the 28,600 refugees resettled in the first half of 2025 [1, 5].

The United Nations High Commissioner for Refugees (UNHCR) has proposed rural resettlement outside major cities as a way for countries to increase their resettlement capacity and, in doing so, distribute refugees more fairly both globally and within their borders [6]. Since 2005, Australian policy has prioritised dispersing refugees away from major cities, aiming to resettle half of the country’s refugees in non-metropolitan areas, known in the Australian vernacular as rural and regional [7, 8]. Similarly, European nations such as the United Kingdom, Ireland, Sweden, Norway, and Germany have implemented policies that distribute refugees across regions and smaller communities [9–11]. Resettlement policies in the United States have also shifted from a focus on large metropolitan gateway cities toward smaller communities and rural areas [12]. While often framed as a strategy for ‘burden sharing’, refugee dispersal can increase national resettlement capacity [6]. For example, when New Zealand increased its refugee intake in 2020, it expanded its programme to include additional regional resettlement locations [13]. Although these dispersal policies have been well explored from a social integration viewpoint, their health impacts require further attention.

Experiences of displacement are powerful determinants of health and wellbeing. Refugees and asylum-seekers have poorer mental and physical health outcomes than the general population [14, 15]. Similarly, rural populations face significant health inequities driven by social and environmental factors, including poverty, inadequate infrastructure, and geographic isolation, compounded by poorer access to healthcare services and chronic health workforce shortages [16–18]. Given that health and healthcare disparities are more pronounced in rural areas, consideration must be given to refugees and asylum-seekers at the intersection of multiple sources of vulnerability [16, 18, 19].

This review aimed to understand the healthcare experiences of refugees and asylum-seekers resettled in rural, regional, and other non-metropolitan areas of HICs. The secondary aim was to identify factors that influence these experiences. No international systematic reviews have yet synthesised evidence on healthcare experiences for this population, highlighting a significant gap given the current emphasis on dispersal policies for refugee resettlement. By prioritising experiential qualitative data, this review aims to be responsive to the perspectives of refugees and asylum-seekers on healthcare and provide insight into the human impact of policy. Understanding and acting on lived experience can improve health services, experiences and outcomes, which are especially important for vulnerable populations at greater risk [20, 21].

## Methods

The Preferred Reporting Items for Systematic Reviews and Meta-analyses (PRISMA) and Enhancing transparency in reporting the synthesis of qualitative research (ENTREQ) guidelines were followed [22, 23]. The protocol was registered with PROSPERO on 11 June 2024 (CRD42024553190).

### Searches

We searched 7 databases from inception to 26 June 2024, with an update on 06 October 2025: Medline(OvidSP)[1946-], Embase(OvidSP)[1974-], PsycINFO(OvidSP)[1806-], CINAHL(EBSCOHOst)[1982-], Social Science Citation Index(Web of Science Core Collection)[1900-], BIOSIS Citation Index(Web of Science) and ASSIA(Proquest)[1987-]. The review question, search strategy, and inclusion criteria were informed by the SPICE framework (Setting, Perspective, Phenomenon of Interest, Comparison, Evaluation) [24]. The search strategy included titles, abstracts, author keywords, and subject headings related to the key concepts of setting and perspective: rural areas, refugees, and asylum seekers. We applied methodological search terms, adapted from a previous search developed by NR, to restrict results to qualitative research and align with the review’s evaluation of healthcare experiences and views (Additional file 1) [25]. We limited searches to English without date limits. NR exported search results to Endnote, where book reviews, conference abstracts, preprints, books, book chapters and theses were excluded. The remaining references were exported to the online platform Covidence, designed to support systematic reviews, for duplicate removal and screening.

### Study selection

Titles and abstracts of articles identified in our search were screened by KW and a second reviewer (RK, GB, CLP, AM, or DK); discrepancies were resolved by a third reviewer. KW and RK or GB screened the full text of potentially relevant articles; discrepancies were resolved through discussion. No automated tools were used for study selection. Inclusion criteria were:

- Original research;
- Refugees and/or asylum-seekers of any age;
- Rural or non-metropolitan areas of an Organisation for Economic Cooperation and Development (OECD) member country;
- Data related to views, experiences, or opinions about health and healthcare services;
- Qualitative analysis or mixed-methods, where qualitative results could be isolated.

OECD membership served as a proxy for HICs; in 2022, 28 of the top 30 HICs hosting refugees and asylum-seekers were OECD members [26]. Since concepts of rurality vary considerably across countries, a setting met our inclusion criteria if it was not a country’s capital and was described as rural, non-metropolitan, or regional (a term commonly used in Australian literature to refer to smaller communities). When this was unclear, the authors were contacted. Mixed populations (rural/urban, patient/provider) were included where the results could be easily separated.

### Data extraction strategy

For included studies, KW recorded author(s), publication year, country, aim, methods, population characteristics, and type of health service in a Covidence extraction table, which was exported to Microsoft Excel. RK and GB checked the data extraction table.

### Study quality assessment

Article quality was appraised using the Critical Appraisal Skills Programme (CASP) Qualitative Research Checklist [27]. KW appraised all papers, with RK or GB providing an independent assessment; discrepancies were resolved through discussion. As in Katsakou and Pistrang [28], the checklist provided a framework for considering each study’s contribution to the review, but was not used to exclude studies.

### Data synthesis

Interpretation of the findings was informed by concepts related to the social determinants of health, including the Dahlgren-Whitehead Rainbow Model [29], which sensitised the research team to the influence of structural, social, environmental, and economic determinants on health experiences and access to care. Data synthesis followed the three stages of thematic synthesis discussed by Thomas and Harden [30]. KW coded all results meaningful to the research question line-by-line in the computer programme NVivo. Initial codes were compared across studies and grouped into descriptive themes that captured recurring patterns and ideas in the data. Through repeated discussion, close reading of coded extracts, and consideration of how the descriptive themes related to the research questions, KW, ST, and CP developed analytical themes. This inductive and iterative approach enabled the generation of higher-level concepts with explanatory power, grounded in participant accounts yet extending findings beyond individual studies.

### Reflexivity

The review team comprised researchers from medical, public health, and social science backgrounds, with several authors (KW, GB, and RK) having prior clinical experience in rural healthcare settings. These perspectives informed the interpretation of the data and the understanding of rural health systems. Regular discussions among the research team, including those between clinical and non-clinical researchers, were used to challenge assumptions, compare interpretations, and refine descriptive and analytical themes. Attention was paid to ensuring that all themes remained grounded in refugee and asylum-seeker accounts and in the narrative findings of the included studies.

## Results

### Review statistics

**Fig. 1:**
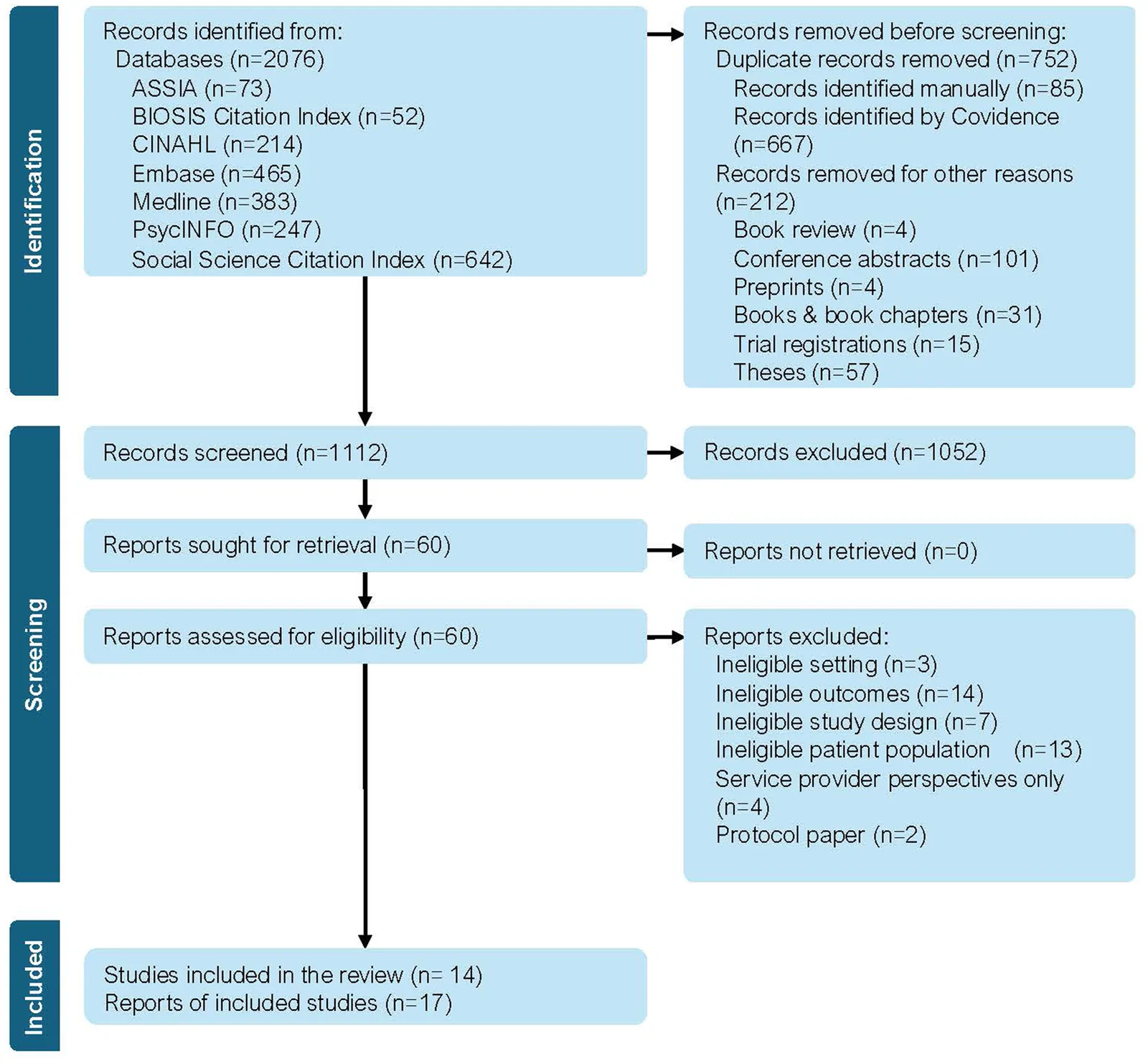
PRISMA flow diagram using the format from Page et al. [22]

Database searches yielded 2076 records; 212 were excluded for format (book reviews, conference abstracts, preprints, books, book chapters, trial registrations, and theses), and 752 were removed as duplicates. After title and abstract screening, 60 full-texts were retrieved and reviewed against the eligibility criteria. Inter-rater reliability for full-text review between KW and RK was substantial (kappa = 0.80, % agreement = 92%) and moderate between KW and GB (kappa = 0.68, % agreement = 86%) [31]. At full-text review, 43 articles were excluded for the following reasons: ineligible setting (*n* = 3); ineligible outcomes (*n* = 14); ineligible study design (*n* = 7); ineligible patient population (*n* = 13); service provider perspectives only (*n* = 4); and protocol paper (*n* = 2) (Fig. 1). Seventeen articles were included.

### Characterisation of included studies

Seventeen articles published in peer-reviewed journals between 2006 and 2025 represented fourteen unique studies from five countries. They included 347 refugees and asylum-seekers in non-metropolitan areas. Ten studies were conducted in Australia [32–44], and single studies were reported from Norway [45], Germany [46], the United States [47], and New Zealand [48]. Twelve were qualitative studies [32, 34–46, 48], and two were mixed-methods studies [33, 47]. All studies included refugee and asylum-seeker perspectives, with five also incorporating views from service providers and/or volunteers [35, 36, 38–40, 46, 48]. Refugees and asylum-seekers originated from Africa, Europe, Asia, and the Middle East. All studies contained qualitative data about health and healthcare experiences. However, in six studies [33–36, 38, 39, 42–44], health and health-related findings were part of a broader exploration of general refugee resettlement experiences rather than being the central focus. Studies collected data using interviews, focus groups, and ethnographic observation. Table 1 presents the characteristics of the included articles.

**Table 1:** Characteristics of included studies

Study	Country	Study aim	Design	Qualitative methods	Participant groups	Refugee and asylum-seeker sample size *	Origin of refugee and asylum-seekers*	Study focus
Au et al., 2021 [32]	Australia	To explore healthcare experiences of refugees in North Queensland, Australia.	Qualitative	Interview	Refugees	14	Congo, Iraq, Myanmar, Somalia	Healthcare
Correa-Velez et al., 2013 [33]	Australia	To examine social exclusion experiences among resettled refugee men in urban and regional Queensland.	Mixed-methods	Interview	Refugees	8	Africa	General resettlement
Eid et al., 2025 [34]	Australia	To understand factors that contribute to belonging among young Karen refugees in regional Victoria.	Qualitative	Interview	Refugees	5	Myanmar	General resettlement and healthcare
Grønseth, 2006 [45]	Norway	To explore how cultural differences shape Tamil refugees’ experiences of health care in Northern Norway.	Qualitative	Ethnographic observation and interview	Refugees	100	Sri Lanka	Healthcare
Hahn et al., 2020 [46]	Germany	To explore the experiences of refugees, health care professionals, and health administrators of refugee health care in a rural area of Germany.	Qualitative	Interview and focus group discussion**	Refugees, health professionals, health administrators	12	Afghanistan, Iraq	Healthcare
Hawkes et al., 2021a † [35] Hawkes et al., 2021b † [36]	Australia	To explore resettlement experience, including factors contributing to mental distress, among women of refugee background resettled in regional Australia.	Qualitative	Interview	Refugees, resettlement service providers and volunteers	9	Not specified	General resettlement and healthcare
Kelley et al., 2024 [47]	United States	To explore health and health-seeking practices of South-East Asian older refugees in the USA, four decades after resettlement.	Mixed-methods	Interview	Refugees	37	Cambodia, Laos	Healthcare
Lloyd et al., 2014 [37]	Australia	To explore how resettling refugees experience a new health environment and develop health literacy practices.	Qualitative	Interview	Refugees	20	Congo, Myanmar, Uganda, Rwanda, Sudan	Healthcare
Shrestha-Ranjit et al., 2020 [48]	New Zealand	To examine the accessibility and acceptability of health promotion services for Bhutanese refugee women in regional New Zealand.	Qualitative	Interview and focus group discussion**	Refugees, health professionals	40	Bhutan	Healthcare
Smith et al., 2019 § [38] Smith et al., 2020 § [39]	Australia	To examine the resettlement experiences and its influencers among former refugees in Launceston, Tasmina, including mental health.	Qualitative	Interview and focus group discussion	Refugees, service providers (health, education and social services)	25	Afghanistan, Bhutan, Iran, Myanmar, Sierra Leone, Sudan	General resettlement and healthcare
Sypek et al., 2008 [40]	Australia	To explore the impact of regional resettlement on rural health services and identify critical health infrastructure needed for refugee support.	Qualitative	Interview	Refugees, healthcare providers and managers, support worker volunteers	7	Not specified	Healthcare
White et al., 2025 [41]	Australia	To explore the experience of refugee access to a multi-disciplinary refugee health team in regional New South Wales.	Qualitative	Interview	Refugees	17	Iraq	Healthcare
Wood et al., 2019 [42]	Australia	To explore how employment and volunteering influence the health and wellbeing of refugees in regional Australia and identify areas for appropriate service provision.	Qualitative	Interview	Refugees	9	North-eastern and West Africa	General resettlement
Ziersch et al., 2020 ‡ [43] Mwanri et al., 2023 ‡ [44]	Australia	To examine how integration factors, social determinants of health, and social ties influence settlement and wellbeing of refugees in a rural South Australian town.	Qualitative	Interview	Refugees	44	Myanmar, Democratic Republic of Congo and surrounding regions	General resettlement and healthcare

* only refugee or asylum-seeker participants living in a rural or regional area

** refugees only included in focus group discussions

† articles form part of the same study

‡ articles form part of the same study

§ articles form part of the same study

### Study quality assessment

Whilst quality assessment using the CASP tool (Additional file 2) did not determine an article’s inclusion in the review, it allowed the research team to consider our confidence in the study and its findings. Papers with more detailed reporting of their methods and which provided thicker, richer descriptions of their results and emerging themes contributed more extensively to theme development. Conversely, articles with a more limited presentation of their methods, data or results contributed less to line-by-line coding and theme development for this review. The CASP domain most frequently lacking was the consideration of researcher-participant relationships and reflexivity (Item 6), which may be due in part to established reporting norms in this field (and possibly due to limitations in the word count associated with journal articles). This means it is difficult to judge whether or how the researchers’ perspectives shaped the generation and interpretation of the findings.

### Data synthesis

Line-by-line coding generated 51 unique codes (Additional file 3). KW developed codes to reflect the meaning and content of each sentence. These codes were then distilled into the following seven descriptive themes summarising the health experiences of refugees and asylum-seekers after resettlement in non-metropolitan areas (Additional file 4):

- Cultural and personal identity shifts after resettlement;
- Displacement affects emotional and psychological wellbeing;
- Language remains a major obstacle long after migration;
- Navigating the health system is complex;
- Care in health settings must be safe, accessible, and culturally appropriate;
- Employment is key to financial security and healthcare access; and
- Housing and transport are particularly challenging in rural areas and shape health and access to care.

**Fig. 2:**
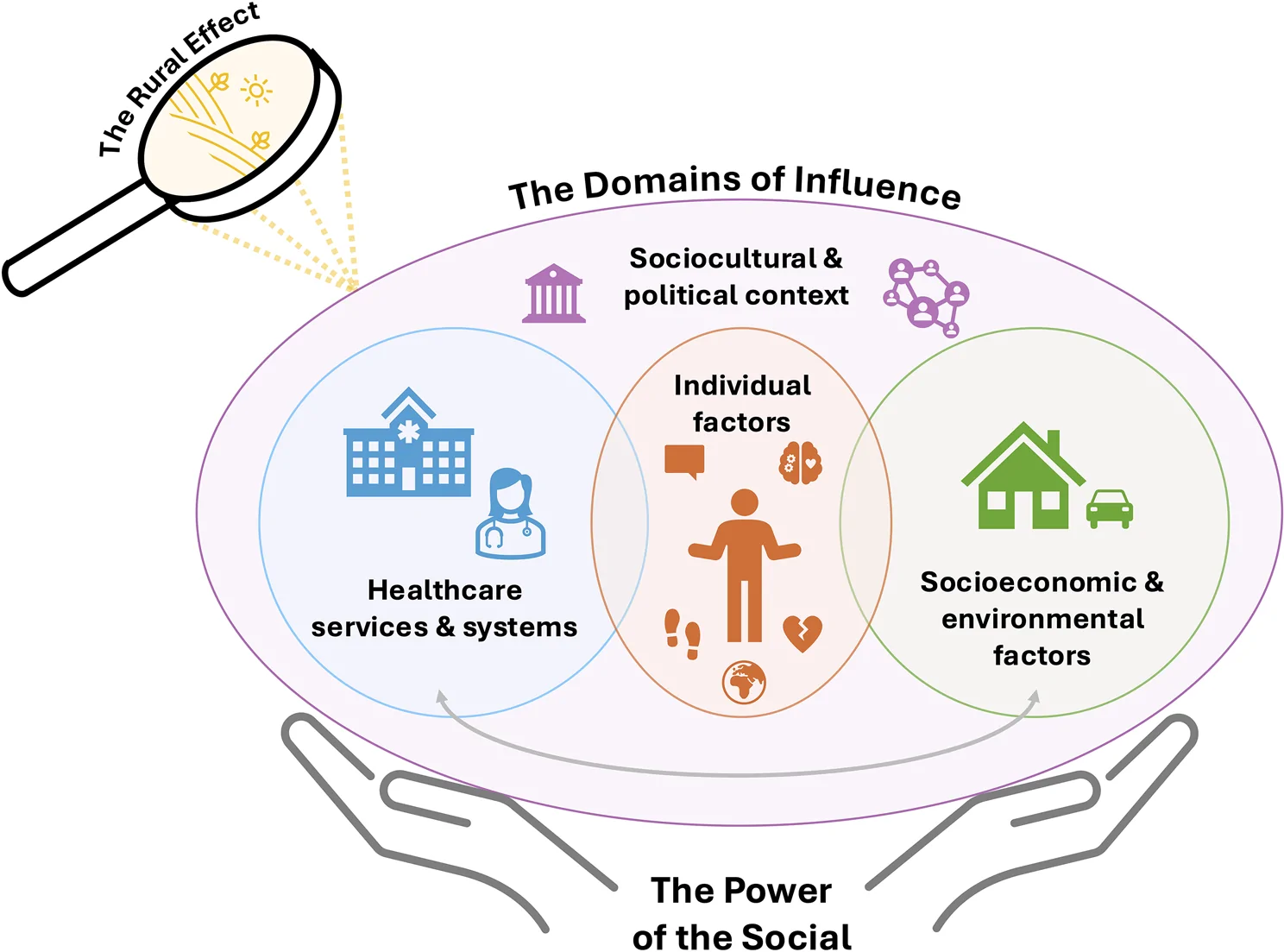
Analytical themes diagram

Descriptive themes were synthesised into three analytical themes that highlight factors that underlie these experiences, presented visually in Fig. 2. Analytical theme 1 is *The Domains of Influence.* This theme explores how individual factors, healthcare services and systems, socioeconomic and environmental factors, and sociocultural and political contexts converge to shape the healthcare experiences of refugees and asylum seekers after rural resettlement. The second analytical theme, which we termed *The Power of the Social* (represented in Fig. 2 by supportive hands), describes the impact of social forces and community on the healthcare experiences of refugees and asylum seekers, particularly in smaller communities where social connectedness, or its absence, becomes more pronounced. *The Rural Effect –* analytical theme 3 – focuses on how rurality acts as a contextual magnifier of both the barriers and enablers of positive healthcare experiences. Figure 2 illustrates how these three themes interact to shape refugee and asylum-seeker healthcare experiences, specifically how factors within *The Domains of Influence* and *The Power of the Social* are contextually modified by rurality (represented by the magnifying glass). The figure also demonstrates how the three themes interact and mutually reinforce one another, recognising that an individual’s health experience is shaped by the convergence of multiple structural, social, and contextual influences rather than any single factor in isolation.

These analytical themes are discussed in more detail below; when presenting extracts from included papers, italicised text reflects first-order quotes from refugees and asylum-seekers (or interpreters), while standard text indicates second-order commentary from article authors.

### The Domains of Influence

#### Individual factors

Healthcare experiences among refugees and asylum-seekers were shaped by a range of individual-level factors, including English proficiency, experiences of trauma and displacement, cultural norms around healthcare and help-seeking, and differing expectations of healthcare systems after resettlement. Among these, English proficiency was the most discussed individual factor, influencing all aspects of a person’s life, including how people navigate and interpret their interactions with health services. Participants described how lacking language skills made everyday healthcare interactions and decision-making challenging [32, 36, 39, 41, 43, 44, 47], and emphasised that learning English was “*the most important thing*” needed to settle and self-advocate [39]. The emotional and practical challenges of limited language skills, including feeling burdensome, misunderstood, and unable to express their needs, were reflected in participant accounts:

*… because they cannot speak English with just brush them off when they say something, you know the little things that people face … they don’t really want to go there again …* [39]

English proficiency, posed specific challenges in rural areas with small populations and limited interpreting resources:

Help-seeking for many Karen young people would involve utilising the services of an interpreter who would likely be known to the young person seeking help *… I share my problem here, and then my problem might get spread. It’s just thing like that. That’s why, ah I don’t want to go there. Because other people might know.* [34]

Refugees were resourceful, recognising days that interpreters were not available and avoided seeking health care on those days *… it was a Friday, there’s no interpreter … so I did not even go to the doctor.* [32]

Past trauma experiences were noted to impact both mental health and how refugees and asylum-seekers engage with health services:

*Because this is the case in my country … a teacher or a doctor or some other officer. They want people to listen only … That is why they always scared of anyone who has position or authority … I remind them, ‘this is not Burma, you feel free to ask …’* [32]

A person’s cultural norms were also a commonly discussed determinant of experiences, particularly if expectations of care and care seeking were misaligned:

*The doctor explained … the type of sickness you have in the long run will not be good for you. In Africa the doctor does not reveal stuff to us … This is not good information for us.* [37]

… participants referred to a specific cultural concept known as ‘ana’ in Burmese. ‘ana’ refers to a desire to avoid burdening others, impacting the willingness of Karen youth to seek out and engage with supports and services … P3(F17) illustrated the concept of ‘ana’ in her own hesitancy to seek support when needed: ‘*I don’t want to share with them … the word is “ana.” I don’t want to be a burden to other people*’. [34]

#### Healthcare services and systems

Beyond the individual, healthcare experiences were shaped by the organisation, accessibility, and cultural responsiveness of local healthcare services and systems. Participants described challenges navigating unfamiliar health systems, communicating with service providers, and accessing culturally safe and inclusive care.

Healthcare services and systems can be confusing and complicated for newcomers, although some services have developed systems that support newly arrived refugees in navigating these systems:

Haymar Aeindra (Burma, woman) spoke of issues understanding how Medicare (the government medical scheme that subsidizes healthcare) worked, which led to her not receiving the rebates she was entitled to. [44]

Participants in this study reported being taken to the medical appointments and having the health environment mediated and information interpreted for them by volunteer and case workers who support this group as they resettle: *They were the ones supporting and helping them, who explained to me how the GP works and what kinds of questions to ask.* [37]

Differences in health beliefs complicated clinical interactions, and a lack of culturally sensitive and inclusive care was described:

*Norwegian doctors don’t understand. The doctor thinks I’m crazy. I have a sore throat, and it aches in my breast. I’m not going to any psychiatrist. I have a problem, and I’m concerned. I’m not crazy.* [45]

Participants spoke of cultural misunderstandings relating to treatment, which adhered to a Westernised medical model *… if they have to take that drug for 2 or 3 times a day and they are devout Muslims, whether they will take those drugs on those days because it means drinking water, and they break their fast.* [38]

Challenges in culturally responsive care also extend to communication between healthcare services and patients. Many patients noted that appointment information and other details would frequently be provided by health services in written English only, which posed difficulties not only for those with limited English but also for those who spoke English proficiently but could not read or write it [34, 44, 47]. This reflects not a mere issue of individual language proficiency, discussed above, but a broader system-level reliance on monolingual, text-based communication that do not respond to patient needs:

In most cases participant reported they were sent appointment information or reminders via SMS (short message service) and sometimes mail, which was challenging … *There were a lot of times I forgot about my appointments so even though they send me like text messages like a reminder, but sometimes it is hard for me to understand.* [41]

Even for older community members who can speak English proficiently, many cannot read and write the language and are thus unable to complete necessary documents to receive medical services. [47]

Despite these challenges, refugees frequently expressed gratitude for the support and medical care provided in their new country. However, it is important to note that some accounts suggested a reluctance or perceived inability to critique services, with participants instead focusing on comparisons to the limited healthcare access they had previously experienced:

*… in my country, you know, growing up in the countryside there is no doctor, there’s no nurse so we have to walk, four hours, six hours. So, in comparison … here is really, really good …* [39]

#### Socioeconomic and environmental factors

Healthcare experiences and access were strongly shaped by socioeconomic and environmental factors, particularly through financial insecurity, employment challenges, housing and transport barriers, and geographic distance from services.

Financial stress frequently limited access to care, as evidenced by a study from the United States where refugees discussed insurance-related health pressures [47]. Even for studies in Australia, where universal healthcare is available, participants reported financial barriers to care when doctors chose not to ‘bulk bill’, the term for a government-funded visit [42, 43]. Moreover, financial barriers to medications and nutritious food remained even where governments funded health services [37, 42]:

*When you are unemployed sometimes you feel like there is something, you need to go and see the doctor … Because you don’t have the money, you feel like you can just keep that disease in you because it’s not too bad.* [42]

Multiple papers referenced how many refugees and asylum-seekers struggled to have pre-settlement education and employment recognised or find skilled employment after completing education in their new country, contributing to financial stress and frustration [33, 42–44, 48]. Community members and individuals found this particularly challenging for people with experience in the health professions who might otherwise be able to provide culturally appropriate care for fellow refugees:

*I worked almost 15 years [as a doctor … As soon as I arrived I] tried to register, tried to get my qualifications recognized. It was too hard, expensive and then too many formalities. I didn’t have the finances to afford that.* [44]

*We have a number of people within our community who have done nursing here. It would be better for us if they could work in our GPs’ clinics … if that happen then they understand our socio-cultural context, which then contributes to make the health services more effective.* [48]

The reality of environmental factors, like geographic distance and urban transport planning, was also an important consideration for participants trying to attend appointments [47]:

The Town has public transport, but this is limited and distances can be quite vast when people need to travel to a neighbouring town or to the city. This was particularly challenging for people who were new to the country and had little option but to walk or catch public transport. [43]

#### Sociocultural and political context

The broader sociocultural and political context of resettlement and rural areas shaped healthcare experiences through community attitudes, discriminatory and inclusive practices, and government policies that affected access to healthcare and social support. Refugees were particularly sensitive to discriminatory and welcoming practices, recognising when community members and healthcare staff were being supportive or dismissive:

*People say that Australia is good that there is no discrimination but it is. It’s underground … I call that submarine discrimination … She didn’t use the word but the way how the body language is talking directly, send a message we don’t like you here.* [42]

*When first we came here, [the nurse] was supporting us. She helped us with all the paperwork and health care records. She arranged the appointments for us, and she wanted to make sure that we got all the support that we needed …* [41]

Government policies were major contributors to healthcare experiences and access. Even in countries with government-funded social welfare, health, and disability services, such as Australia and Germany, refugees reported feeling disadvantaged and, in some cases, excluded from services or experiencing delays in accessing care due to strict eligibility requirements:

*If I compare my life with the other Australians in fact we are the last. We are in the bottom class of Australia.* [33]

*… it’s a bit difficult, because everything has to be approved first, then you go through social services, then the district, then, for example, a health insurer, then the hospital; and he says it takes time to get anything done eventually.* [46]

### The Power of the Social

Across the studies, social factors were highlighted as critical determinants shaping refugee and asylum-seeker experiences of healthcare. Following resettlement, both formal and informal networks developed among new arrivals, host communities, settlement service providers, local migrant communities, and faith-based organisations. These networks eased the transition into a new country and facilitated engagement with health services through knowledge sharing and practical support, like transportation:

*So initially it was other people who came here before us, it is the community people who arrived here before us, they were very helpful and explaining things and telling us this is what we do here.* [41]

When Emmanuel needed to go to Adelaide for a family health emergency, he had not been there before, but fortunately a fellow member of the Catholic Church volunteered to drive him there. [43]

Social supports fostered a sense of belonging and helped counter isolation, whilst providing opportunities to combat barriers to care that exist within the healthcare system. For example, friends, family, and fellow refugees frequently provided language support when interpreter services were unavailable [37, 41, 44]. However, an over-reliance on community support, especially from fellow migrants, can strain individuals, their families, and the broader community:

Due to the lack of official translation services offered by health-care facilities, older refugees rely on local community or family members to translate for them. [47]

*Most of my time is spent for taking people to appointments … so I have to take time from my TAFE* [English classes] *to take someone to the appointment, bring them back, take the other one.* [41]

### The Rural Effect

For refugees and asylum-seekers, rurality acted as a contextual modifier of healthcare experiences through both supportive and exclusionary mechanisms. Enhanced service proximity, perceptions of community safety, and opportunities for care coordination coexisted with transport barriers, workforce shortages, limited service availability, and interpreter scarcity. Participants recognised these tensions, with some weighing the trade-offs of remaining in rural communities against relocating to metropolitan centres with greater service availability and support infrastructure.

Refugees acknowledged that living in smaller, non-metropolitan areas sometimes enhanced service proximity [32], information sharing, and care coordination [41], thereby improving their healthcare experiences.

The proximity of services facilitated attending multiple appointments in contrast to metropolitan cities *… I can attend five different appointments. Other people who live in other cities told me that we cannot attend more than one appointment per day because they’re busy on the road …* [32]

Smaller communities were also seen to provide safety and security, contributing to a sense of wellbeing in their new rural home:

*[The Town] is a good place because it’s a place where you can live with your kids in a good condition than big cities … Even friends from there [other cities], they always tell us that ‘the place where you live, it’s a very, very good place to keep safe your kids for their small age.* [43]

In some rural communities, specialised services and volunteer networks coordinated care processes and helped mitigate the geographic and structural barriers facing refugees and asylum-seekers, thereby improving healthcare access and experiences:

*Yes, we were planning to go to Newcastle (metropolitan city) to see some specialists, but they arranged for us to have the appointment in Tamworth (regional city) with a visiting specialist.* [41]

Upon arrival Kelsy is received by case workers and introduced to volunteers from a faith-based group who will assist with resettlement. In the first few weeks, medical arrangements are made on her behalf, and she is taken to appointments … Caseworkers and volunteers tell her about how the health system works and what sort of government assistance she can receive. [37]

However, healthcare barriers also intensified for refugees in rural areas. Participants described long wait times, further complicated by inconsistent access to interpreter services, and limited options for health providers:

Participants living in regional areas reported higher levels of dissatisfaction with getting help in times of medical emergency. They reported that only a small number of general practitioners provided services to refugee families … and the absence of an interpreter as common barriers to accessing primary healthcare services. [33]

… the service centers in the towns were quite small, often with only one or two members of staff, as such supports provided as well as information about the available supports through these services were limited … [44]

Transportation emerged as a widely recognised challenge affecting both daily life and healthcare access for refugees and asylum-seekers in non-metropolitan areas. Difficulties included limited public transport infrastructure and the need to travel to larger cities for specialised treatment, which was often complex and costly [40, 43, 44]:

*Just need help to take go to the hospital, but no bus. No nothing over here.* [47]

*The first year was very difficult for me, I did not have a car and I was taking care of six kids. Sometimes when they had an appointment, I needed to walk to the school to get them from school and then walk to the appointment. Sometimes it was raining, and the weather was very cold, it was just very difficult to walk through the weather.* [41]

… another refugee was told he would have to travel to Sydney for treatment, a trip he could not afford. [40]

The benefits of smaller rural and regional communities did not always outweigh the challenges and limited services, and some chose to relocate:

*Everybody loves to live here because it is a very good city but because of the shortage of interpreters, everyone is running away … Most of my friends who move to Brisbane, they told me … everywhere you go, there is a face to face interpreter.* [32]

*Many of my friends, they say, I’m not supported. I’m not feel – Like this isn’t the right environment for me’ and then they move to somewhere else.* [36]

## Discussion

This systematic review explored healthcare experiences among refugees and asylum-seekers in non-metropolitan areas of high-income countries. Through thematic synthesis, we developed three analytical themes that lie beneath care experiences:

- *The Domains of Influence*;
- *The Power of the Social*; and
- *The Rural Effect*.

Most eligible studies were from Australia, reflecting policymakers’ and government efforts to resettle refugees outside major cities, as well as the country’s focus on rural health research. Nevertheless, the two populations resettled the longest ago were from Northern Norway [45] and coastal Alabama [47], reinforcing that Australia is neither the only nor the first country to implement rural resettlement strategies. While healthcare systems and rural contexts will vary across countries, findings were broadly consistent across studies, including those conducted outside Australia. This suggests that the themes identified in the synthesis may be relevant to other high-resource countries that employ rural- or dispersal-based refugee resettlement policies.

*The Domains of Influence* identified in this review as shaping refugee and asylum-seeker healthcare experiences correspond with established social determinants of health that contribute to health inequities [49, 50]. Language proficiency was a key factor, consistent with the evidence that language is a major social determinant of health for immigrant communities [51]. While this review focuses on non-metropolitan settings, these social and structural determinants are also recognised as relevant to refugees and asylum-seekers in urban areas [52]. However, as explored in *The Rural Effect* theme, our synthesis suggests that in rural settings these challenges may be intensified by geographic isolation, limited specialist services, workforce and interpreter shortages, and transport barriers. This emphasises the need for refugee resettlement and rural health policies that address upstream social determinants of health while also strengthening rural healthcare infrastructure, transport access, and interpreting capacity.

Employment represents a critical nexus through which social determinants shape healthcare experiences and access to care. Non-recognition of vocational skills limits employment opportunities for individuals, contributes to financial strain, and may adversely affect the healthcare experiences of their broader community for people who would otherwise be employed in health roles [43, 44, 48]. Evidence suggests that clinicians from the same racial and ethnic background as patients can improve communication and patient satisfaction [53, 54], thereby amplifying the effects of underemployment among refugee populations. While some larger cities have implemented programs to assist refugee health professionals in obtaining local registration with good effect [55, 56], similar initiatives are lacking in rural settings and should be a priority for local policymakers and employment providers.

Although resource constraints continue to challenge non-metropolitan settings, the development of culturally responsive, locally tailored services remains critical. This review highlights that discrimination and systemic racism persist within the rural healthcare continuum, from administrative processes to patient-provider interactions. These issues are best exemplified by the persistent use of English-only appointment reminders for patients with limited English proficiency [41, 44]. For people with no or limited English, interpreters are vital to providing the best possible quality care [57]. For refugees, who are inequitably positioned within host populations, addressing barriers to healthcare interpretation and culturally appropriate care is an even greater concern [58]. Ensuring timely access to interpreter services and comprehensive training in culturally safe and responsive care for staff should therefore be key priorities for rural health services and professionals.

The analytical theme, *The Power of the Social*, reflects how relationships and social networks affect healthcare experiences. Social capital is a multidimensional sociological concept that describes how relationships, trust, social cohesion, and civic engagement serve as productive resources that support the goals and needs of individuals and groups [59, 60]. From information-seeking to clinical encounters, refugees and asylum-seekers rely heavily on social capital, often through connections with ethnic or national peers, to navigate unfamiliar services, share knowledge, and bridge gaps in care. This aligns with evidence that social capital can mitigate the adverse effects of social determinants on health, particularly for disadvantaged communities and minority populations [61]. Additionally, connections among refugee community members can positively impact both integration and health outcomes [62, 63]. In rural contexts, the smaller size and geographic dispersal of immigrant populations may limit opportunities to build social networks. Policies that promote the co-location of refugees and asylum-seekers from similar backgrounds may strengthen the protective influence of social capital in these communities, thereby improving healthcare experiences [44, 64].

Highlighting how rural life can both enhance and hinder refugees’ healthcare experiences is a novel contribution of this review. This review suggests that rurality does not function as an isolated determinant of healthcare experiences. Rurality instead magnifies the existing social and structural determinants of health for refugees and asylum-seekers, which we termed here *The Rural Effect*. This theme also operates through mechanisms such as interpreter scarcity, provider shortages, transport barriers, and geographic isolation. At the same time, smaller, non-metropolitan communities may facilitate service integration, care coordination, and interpersonal connectedness. These dynamics emphasise the complex and context-dependent nature of rural healthcare experiences for refugees and asylum-seekers navigating life in a new country. Framing rurality as a contextual modifier aligns with existing literature documenting poorer health outcomes and service challenges in non-metropolitan areas [16, 18], while recognising that other aspects of wellbeing, such as happiness and life satisfaction, can be higher among rural communities in HICs [65, 66].

This synthesis demonstrates how the supportive and exclusionary dimensions of rural life are experienced specifically by resettled refugees and asylum-seekers. Refugee integration literature has highlighted the importance of social connectedness for successful integration, including better physical and mental health after resettlement [64], while rural health research has long emphasised the impact of structural, workforce, and resource barriers on population health in non-metropolitan communities [17, 18]. By bringing these bodies of literature together, this review illustrates how refugees and asylum-seekers experience these tensions simultaneously within rural resettlement contexts. For policymakers and service providers, capitalising on the strengths of smaller communities – for example, through service co-location or inter-provider collaborations that promote continuity of care – may be as important as addressing the known structural and resource limitations of rural areas.

Just as healthcare experiences are shaped by the convergence of multiple factors, a multifaceted approach is essential for improving the healthcare experiences of refugees and asylum seekers in non-metropolitan areas. The findings relating to *The Domains of Influence* highlight the importance of addressing upstream social and structural determinants of health through policies that support employment opportunities, affordable transport, culturally responsive healthcare, and interpreter access. The findings associated with *The Power of the Social* suggest that strengthening community connectedness and social capital should remain central to rural resettlement planning, including through community health workers, partnerships with faith-based organisations, peer-led support initiatives, and policies promoting co-location of refugees and asylum-seekers from similar backgrounds [64, 67]. Finally, *The Rural Effect* focuses on the need for rural health systems planning that recognises how workforce shortages, geographic isolation, and limited service availability may disproportionately affect resettled refugees and asylum-seekers. Strategies such as service co-location, improved transport access, inter-provider collaboration, and investment in rural interpreting infrastructure may therefore be particularly important within non-metropolitan resettlement settings. Collectively, these findings emphasise that improving healthcare experiences for refugees and asylum-seekers requires coordinated action across health systems, resettlement policy, and rural community development

### Strengths and limitations

To our knowledge, this is the first systematic review to explore healthcare experiences from the perspective of refugees and asylum-seekers resettled in non-metropolitan areas of HICs. We intentionally focused on experiential data provided by refugees and asylum-seekers, centring patient and community voices as a critical step toward improving health service delivery [21]. The inclusion of only peer-reviewed journal articles strengthens the quality of the evidence, but may have excluded insights from relevant grey literature [68]. Furthermore, as with other thematic syntheses of qualitative data, the review only analysed data reported within the studies’ publications, potentially omitting important background details or unpublished findings. As identified during quality appraisal, the included articles varied in reporting quality, particularly regarding reflexivity and researcher-participant relationships. Limited discussion of these issues reduced transparency regarding how findings were generated and interpreted within some studies. Future qualitative research in this area should more explicitly reflect and report on potential sources of bias and researcher influence to strengthen the credibility of findings.

The multidisciplinary review team, comprising nine academics from the medical and social sciences, ensured comprehensive literature searching, cross-checking, and synthesis. Notably, three authors (KW, GB, and RK) have clinical experience in rural healthcare across various regions of Australia, which enhanced the contextual interpretation of the findings. These experiences may also have shaped the synthesis, particularly given the predominance of Australian studies. However, the synthesis was undertaken collaboratively by KW, ST, and CP, who brought both clinical and social science perspectives to the analysis, ensuring that interpretations were refined through multidisciplinary discussion rather than shaped solely by clinical perspectives in rural healthcare.

Excluding non-English-language articles may have omitted relevant research published in other languages. Still, the synthesis included two studies conducted in non-English-speaking European countries [45, 46]. These studies reported broadly consistent findings with those from North America and Australasia, supporting the relevance of the synthesis themes beyond English-speaking settings. Nevertheless, some limits exist. For example, the review used a broad definition of non-metropolitan settings to account for international variation in definitions of rurality. This may have introduced variation in the healthcare environments and rural communities represented in the synthesis. Healthcare systems, resettlement policies, interpreting infrastructure, and rural service provision vary across and within countries, even among highly resourced HICs. As a result, the synthesis should be seen as reflecting diverse non-metropolitan contexts, rather than a single rural experience. While most studies originated from Australia, findings were largely aligned across studies in different national contexts, suggesting that Australian studies contributed greater depth to the synthesis rather than fundamentally distinct findings. Together, these factors suggest that the findings may have particular transferability to comparable high-resource non-metropolitan resettlement settings. Further research across a broader range of linguistic, cultural, geographic, and policy contexts is needed, particularly in countries that employ dispersal policies, where local evidence remains limited.

Given the need to expand resettlement pathways for refugees and the potential of rural resettlement policies to increase host-country capacity [6], research on the health and healthcare impacts of rural resettlement should remain a priority. While all studies included in this synthesis contained health-related findings, several focused on general resettlement experiences. Future research should therefore prioritise longitudinal and prospective studies with an explicit healthcare focus to better characterise the short- and long-term impacts of dispersal policies on the health, healthcare access, and wellbeing of refugees and asylum-seekers.

## Conclusion

In conclusion, enhancing healthcare experiences for refugees and asylum seekers in non-metropolitan areas requires a multidimensional, sustained approach. Health services and providers should balance immediate service improvements with long-term structural reforms that address systemic inequities. Beyond policymakers and service providers, rural communities are critical stakeholders who should proactively embed inclusion and cultural responsiveness in rural resettlement. Strengthening community connections and addressing discrimination are critical enablers of equitable access and positive healthcare experiences. Yet ultimately, ongoing policy attention and investment in rural health systems and workforces are crucial to ensuring positive healthcare experiences for refugees and asylum seekers and to offering a pathway towards more equitable and inclusive rural communities.

## Electronic supplementary material

Below is the link to the electronic supplementary material.

Supplementary Material 1

Supplementary Material 2

Supplementary Material 3

Supplementary Material 4

## Data Availability

Data sharing is not applicable to this article as the information analysed in this study was obtained from published articles included in the reference list. Search strings are available in additional information files.

## Abbreviations

CASP: Critical Appraisal Skills Programme
ENTREQ: Enhancing Transparency in Reporting the Synthesis of Qualitative Research
HICs: High-Income Countries
LMICs: Low- and Middle-Income Countries
OECD: Organisation for Economic Cooperation and Development
PRISMA: Preferred Reporting Items for Systematic Reviews and Meta-Analyses
UNHCR: United Nations High Commissioner for Refugees
