# Supplementary material for: Healthcare experiences of refugees and asylum-seekers resettled in non-metropolitan high-income settings: a systematic review and thematic synthesis of qualitative evidence
Source: Int J Equity Health. 2026 Jul 14;25:238. doi: 10.1186/s12939-026-02933-9 (PMC13651060; doi:10.1186/s12939-026-02933-9)
Supplement: Supplementary file 1 [file 12939_2026_2933_MOESM1_ESM.docx]

# Additional File 1: Search report and search strategy

### Search report

| **Database** | **Platform** | **Coverage** | **Hits: 26/06/2024** | **Hits: 06/10/2025** | **Total:** |
| --- | --- | --- | --- | --- | --- |
| ASSIA | Proquest | 1987-present | 66 | 7 | 73 |
| BIOSIS Citation Index | Web of Science |  | 46 | 6 | 52 |
| CINAHL | EBSCOHost | 1982-present | 181 | 33 | 214 |
| Embase | OvidSP | 1974-present | 360 | 105 | 465 |
| Medline | OvidSP | 1946-present | 311 | 72 | 383 |
| PsycINFO | OvidSP | 1806-present | 217 | 30 | 247 |
| Social Science Citation Index | Web of Science Core Collection | 1900-present | 538 | 104 | 642 |

### Search in ASSIA (Proquest)

#### Date: 26/06/2024

| Set#: S4 | Searched for: (TI,AB,IF("patient satisfaction") AND (TI,AB,IF(survey?) OR TI,AB,IF(questionnaire?))) OR (TI(satisfaction?) OR TI(perce*) OR TI(experience?) OR TI(attitude?) OR TI(view*) OR TI(opinion?) OR TI(preference?) OR TI(expect*) OR TI(challenge?) OR TI(issue*) OR TI(barrier?) OR TI(obstacle?) OR TI(problem*) OR TI(involvement) OR TI(access*) OR TI(engagement) OR (TI(cultur*) NEAR/3 (TI(competen*) OR TI(safe*) OR TI(appropriate*) OR TI(sensitiv*))) OR TI(communication*) OR TI(language*) OR TI(interpreter?) OR TI(enabler?) OR TI(facilita*) OR TI(promoter?) OR TI(opportunit*) OR TI(advantage?) OR TI(disadvantage?)) OR TI(qualitative) OR (AB(qualitative) NEAR/3 (AB(stud*) OR AB(data) OR AB(finding?) OR AB(evaluation) OR AB(analysis))) OR (TI,AB,IF(interview*) OR TI,AB,IF(("focus group" OR "focus groups")) OR ((TI,AB,IF(unstructured) OR TI,AB,IF(semi-structured) OR TI,AB,IF(semistructured) OR TI,AB,IF(open)) NEAR/2 TI,AB,IF(question*)) OR TI,AB,IF(("case students" OR "case studies" OR "case study"))) OR (TI,AB,IF("grounded theory") OR TI,AB,IF(ethnograph*) OR TI,AB,IF(phenomenolog*) OR TI,AB,IF(ipa) OR TI,AB,IF(narratives) OR TI,AB,IF("narrative analysis") OR TI,AB,IF(themes) OR TI,AB,IF("thematic analysis") OR TI,AB,IF(NVivo)) OR (TI,AB,IF(("mixed method" OR "mixed methods")) OR TI,AB,IF("multi* method*") OR TI,AB,IF(multimethod*) OR TI,AB,IF("mixed stud*")) OR ((TI,AB,IF(qualitative) NEAR/2 TI,AB,IF(review)) OR TI,AB,IF(metaethnograph*) OR TI,AB,IF(meta-ethnograph*) OR TI,AB,IF(metasynthes*) OR TI,AB,IF(meta-synthes*)) |
| --- | --- |
| Set#: S5 | Searched for: (TI,AB,IF(refugee?) OR TI,AB,IF("asylum seeker?") OR (TI,AB,IF(humanitarian) NEAR/2 (TI,AB,IF(entrant?) OR TI,AB,IF(migrant?) OR TI,AB,IF(immigrant?))) OR (TI,AB,IF(displaced) NEAR/3 (TI,AB,IF(person?) OR TI,AB,IF(people) OR TI,AB,IF(population?))) OR ((TI,AB,IF(migrant?) OR TI,AB,IF(immigrant?)) NEAR/3 (TI,AB,IF(forced) OR TI,AB,IF(cris?s) OR TI,AB,IF(disaster*) OR TI,AB,IF(war?) OR TI,AB,IF(conflict*) OR TI,AB,IF(persecut*) OR TI,AB,IF(escap*) OR TI,AB,IF(flee*) OR TI,AB,IF(evacuat*) OR TI,AB,IF(political)))) |
| Set#: S6 | Searched for: (TI,AB,IF(rural*) OR TI,AB,IF(regional) OR TI,AB,IF(non-urban) OR TI,AB,IF(non-metro*) OR TI,AB,IF(coastal) OR TI,AB,IF(outport?) OR ((TI,AB,IF(remote) OR TI,AB,IF(isolated)) NEAR/3 (TI,AB,IF(area?) OR TI,AB,IF(communit*) OR TI,AB,IF(local*) OR TI,AB,IF(population?) OR TI,AB,IF(region?) OR TI,AB,IF(settlement?) OR TI,AB,IF(setting?) OR TI,AB,IF(town?) OR TI,AB,IF(village?)))) |
| Set#: S7 | Searched for: [S4] AND [S5] AND [S6] |

#### Date: 06/10/2025

| Set#: S1 | Searched for: (TI,AB,IF("patient satisfaction") AND (TI,AB,IF(survey?) OR TI,AB,IF(questionnaire?))) OR (TI(satisfaction?) OR TI(perce*) OR TI(experience?) OR TI(attitude?) OR TI(view*) OR TI(opinion?) OR TI(preference?) OR TI(expect*) OR TI(challenge?) OR TI(issue*) OR TI(barrier?) OR TI(obstacle?) OR TI(problem*) OR TI(involvement) OR TI(access*) OR TI(engagement) OR (TI(cultur*) NEAR/3 (TI(competen*) OR TI(safe*) OR TI(appropriate*) OR TI(sensitiv*))) OR TI(communication*) OR TI(language*) OR TI(interpreter?) OR TI(enabler?) OR TI(facilita*) OR TI(promoter?) OR TI(opportunit*) OR TI(advantage?) OR TI(disadvantage?)) OR TI(qualitative) OR (AB(qualitative) NEAR/3 (AB(stud*) OR AB(data) OR AB(finding?) OR AB(evaluation) OR AB(analysis))) OR (TI,AB,IF(interview*) OR TI,AB,IF(("focus group" OR "focus groups")) OR ((TI,AB,IF(unstructured) OR TI,AB,IF(semi-structured) OR TI,AB,IF(semistructured) OR TI,AB,IF(open)) NEAR/2 TI,AB,IF(question*)) OR TI,AB,IF(("case students" OR "case studies" OR "case study"))) OR (TI,AB,IF("grounded theory") OR TI,AB,IF(ethnograph*) OR TI,AB,IF(phenomenolog*) OR TI,AB,IF(ipa) OR TI,AB,IF(narratives) OR TI,AB,IF("narrative analysis") OR TI,AB,IF(themes) OR TI,AB,IF("thematic analysis") OR TI,AB,IF(NVivo)) OR (TI,AB,IF(("mixed method" OR "mixed methods")) OR TI,AB,IF("multi* method*") OR TI,AB,IF(multimethod*) OR TI,AB,IF("mixed stud*")) OR ((TI,AB,IF(qualitative) NEAR/2 TI,AB,IF(review)) OR TI,AB,IF(metaethnograph*) OR TI,AB,IF(meta-ethnograph*) OR TI,AB,IF(metasynthes*) OR TI,AB,IF(meta-synthes*)) |
| --- | --- |
| Set#: S2 | (TI,AB,IF(rural*) OR TI,AB,IF(regional) OR TI,AB,IF(non-urban) OR TI,AB,IF(non-metro*) OR TI,AB,IF(coastal) OR TI,AB,IF(outport?) ) OR ((TI,AB,IF(remote) OR TI,AB,IF(isolated)) NEAR/3 (TI,AB,IF(area?) OR TI,AB,IF(communit*) OR TI,AB,IF(local*) OR TI,AB,IF(population?) OR TI,AB,IF(region?) OR TI,AB,IF(settlement?) OR TI,AB,IF(setting?) OR TI,AB,IF(town?) OR TI,AB,IF(village?))) |
| Set#: S3 | TI,AB,IF(refugee?) OR TI,AB,IF("asylum seeker?") OR (TI,AB,IF(humanitarian) NEAR/2 (TI,AB,IF(entrant?) OR TI,AB,IF(migrant?) OR TI,AB,IF(immigrant?))) OR (TI,AB,IF(displaced) NEAR/3 (TI,AB,IF(person?) OR TI,AB,IF(people) OR TI,AB,IF(population?))) OR ((TI,AB,IF(migrant?) OR TI,AB,IF(immigrant?)) NEAR/3 (TI,AB,IF(forced) OR TI,AB,IF(cris?s) OR TI,AB,IF(disaster*) OR TI,AB,IF(war?) OR TI,AB,IF(conflict*) OR TI,AB,IF(persecut*) OR TI,AB,IF(escap*) OR TI,AB,IF(flee*) OR TI,AB,IF(evacuat*) OR TI,AB,IF(political))) |
| Set#: S4 | [S1] AND [S2] AND [S3] |
| Set#: S11 | ([S1] AND [S8] AND [S9]) AND pd(20240101-20251231) |

### Search in BIOSIS Citation Index (Web of Science)

#### Date: 26/04/2024

| 1 | (TI=(satisfaction$ OR perce* OR experience$ OR attitude$ OR view* OR opinion$ OR preference$ OR expect* OR challenge$ OR issue* OR barrier$ OR obstacle$ OR problem* OR involvement OR access* OR engagement OR (cultur* NEAR/3 (competen* OR safe* OR appropriate* OR sensitiv*)) OR communication* OR language* OR interpreter$ OR enabler$ OR facilita* OR promoter$ OR opportunit* OR advantage$ OR disadvantage$) AND TS=(survey$ OR questionnaire$)) OR TI=qualitative OR AB=(qualitative NEAR/3 (stud* OR data OR finding$ OR evaluation OR analysis)) OR TS=(interview* OR "focus group*" OR ((unstructured OR semi-structured OR semistructured OR open) NEAR/2 question*) OR "case stud*") OR TS=("grounded theory" OR ethnograph* OR phenomenolog* OR ipa OR narratives OR "narrative analysis" OR themes OR "thematic analysis" OR NVivo) OR TS=("mixed method*" OR "multi* method*" OR multimethod* OR "mixed stud*") OR TS=((qualitative NEAR/2 review) OR metaethnograph* OR meta-ethnograph* OR metasynthes* OR meta-synthes*) |
| --- | --- |
| 2 | TS=(refugee$ OR "asylum seeker$" OR (humanitarian NEAR/2 (entrant$ OR migrant$ OR immigrant$)) OR (displaced NEAR/3 (person$ OR people OR population$)) OR ((migrant$ OR immigrant$) NEAR/3 (forced OR cris$s OR disaster* OR war$ OR conflict* OR persecut* OR escap* OR flee* OR evacuat* OR political))) |
| 3 | TS=(rural* OR regional OR non-urban OR non-metro* OR coastal OR outport$ OR ((remote OR isolated) NEAR/3 (area$ OR communit* OR local* OR population$ OR region$ OR settlement$ OR setting$ OR town$ OR village$))) |
| 4 | #3 AND #2 AND #1 |
| 5 | #3 AND #2 AND #1 and English (Languages) |

#### Date: 06/10/2025

| 1 | (TI=(satisfaction$ OR perce* OR experience$ OR attitude$ OR view* OR opinion$ OR preference$ OR expect* OR challenge$ OR issue* OR barrier$ OR obstacle$ OR problem* OR involvement OR access* OR engagement OR (cultur* NEAR/3 (competen* OR safe* OR appropriate* OR sensitiv*)) OR communication* OR language* OR interpreter$ OR enabler$ OR facilita* OR promoter$ OR opportunit* OR advantage$ OR disadvantage$) AND TS=(survey$ OR questionnaire$)) OR TI=qualitative OR AB=(qualitative NEAR/3 (stud* OR data OR finding$ OR evaluation OR analysis)) OR TS=(interview* OR "focus group*" OR ((unstructured OR semi-structured OR semistructured OR open) NEAR/2 question*) OR "case stud*") OR TS=("grounded theory" OR ethnograph* OR phenomenolog* OR ipa OR narratives OR "narrative analysis" OR themes OR "thematic analysis" OR NVivo) OR TS=("mixed method*" OR "multi* method*" OR multimethod* OR "mixed stud*") OR TS=((qualitative NEAR/2 review) OR metaethnograph* OR meta-ethnograph* OR metasynthes* OR meta-synthes*) |
| --- | --- |
| 2 | TS=(refugee$ OR "asylum seeker$" OR (humanitarian NEAR/2 (entrant$ OR migrant$ OR immigrant$)) OR (displaced NEAR/3 (person$ OR people OR population$)) OR ((migrant$ OR immigrant$) NEAR/3 (forced OR cris$s OR disaster* OR war$ OR conflict* OR persecut* OR escap* OR flee* OR evacuat* OR political))) |
| 3 | TS=(rural* OR regional OR non-urban OR non-metro* OR coastal OR outport$ OR ((remote OR isolated) NEAR/3 (area$ OR communit* OR local* OR population$ OR region$ OR settlement$ OR setting$ OR town$ OR village$))) |
| 4 | #3 AND #2 AND #1 |
| 5 | #3 AND #2 AND #1 Timespan: 2024-01-01 to 2025-12-31 |
| 6 | #3 AND #2 AND #1 and English (Languages) Timespan: 2024-01-01 to 2025-12-31 |

### Search in CINAHL (EBSCOHost)

#### Date: 26/06/2024

| S11 | S5 AND S6 AND S9 Limiters - English Language |
| --- | --- |
| S10 | S5 AND S6 AND S9 |
| S9 | S7 OR S8 |
| S8 | ((TI rural* OR AB rural* OR SU rural*) OR (TI regional OR AB regional OR SU regional) OR (TI non-urban OR AB non-urban OR SU non-urban) OR (TI non-metro* OR AB non-metro* OR SU non-metro*) OR (TI coastal OR AB coastal OR SU coastal) OR (TI outport# OR AB outport# OR SU outport#) OR (((TI remote OR AB remote OR SU remote) OR (TI isolated OR AB isolated OR SU isolated)) N3 ((TI area# OR AB area# OR SU area#) OR (TI communit* OR AB communit* OR SU communit*) OR (TI local* OR AB local* OR SU local*) OR (TI population# OR AB population# OR SU population#) OR (TI region# OR AB region# OR SU region#) OR (TI settlement# OR AB settlement# OR SU settlement#) OR (TI setting# OR AB setting# OR SU setting#) OR (TI town# OR AB town# OR SU town#) OR (TI village# OR AB village# OR SU village#)))) |
| S7 | (MH "Hospitals, Rural") OR (MH "Rural Population") OR (MH "Rural Health") OR (MH "Rural Health Centers") OR (MH "Rural Health Services") OR (MH "Rural Areas") OR (MH "Rural Nursing") OR (MH "Rural Health Personnel") OR (MH "Rural Nurses") |
| S6 | TX ((refugee# OR "asylum seeker#" OR (humanitarian N2 (entrant# OR migrant# OR immigrant#)) OR (displaced N3 (person# OR people OR population#)) OR ((migrant# OR immigrant#) N3 (forced OR cris#s OR disaster* OR war# OR conflict* OR persecut* OR escap* OR flee* OR evacuat* OR political)))) |
| S5 | S1 OR S2 OR S3 OR S4 |
| S4 | (TI qualitative OR SU qualitative) OR ((AB qualitative) N3 ((AB stud*) OR (AB data) OR (AB finding#) OR (AB evaluation) OR (AB analysis))) OR ((TI interview* OR AB interview* OR SU interview*) OR (TI "focus group*" OR AB "focus group*" OR SU "focus group*") OR (((TI unstructured OR AB unstructured OR SU unstructured) OR (TI semi-structured OR AB semi-structured OR SU semi-structured) OR (TI semistructured OR AB semistructured OR SU semistructured) OR (TI open OR AB open OR SU open)) N2 (TI question* OR AB question* OR SU question*)) OR (TI "case stud*" OR AB "case stud*" OR SU "case stud*")) OR ((TI "grounded theory" OR AB "grounded theory" OR SU "grounded theory") OR (TI ethnograph* OR AB ethnograph* OR SU ethnograph*) OR (TI phenomenolog* OR AB phenomenolog* OR SU phenomenolog*) OR (TI ipa OR AB ipa OR SU ipa) OR (TI narratives OR AB narratives OR SU narratives) OR (TI "narrative analysis" OR AB "narrative analysis" OR SU "narrative analysis") OR (TI themes OR AB themes OR SU themes) OR (TI "thematic analysis" OR AB "thematic analysis" OR SU "thematic analysis") OR (TI NVivo OR AB NVivo OR SU NVivo)) OR ((TI "mixed method*" OR AB "mixed method*" OR SU "mixed method*") OR (TI "multi* method*" OR AB "multi* method*" OR SU "multi* method*") OR (TI multimethod* OR AB multimethod* OR SU multimethod*) OR (TI "mixed stud*" OR AB "mixed stud*" OR SU "mixed stud*")) OR (((TI qualitative OR AB qualitative OR SU qualitative) N2 (TI review OR AB review OR SU review)) OR (TI metaethnograph* OR AB metaethnograph* OR SU metaethnograph*) OR (TI meta-ethnograph* OR AB meta-ethnograph* OR SU meta-ethnograph*) OR (TI metasynthes* OR AB metasynthes* OR SU metasynthes*) OR (TI meta-synthes* OR AB meta-synthes* OR SU meta-synthes*)) |
| S3 | ((TI satisfaction#) OR (TI perce*) OR (TI experience#) OR (TI attitude#) OR (TI view*) OR (TI opinion#) OR (TI preference#) OR (TI expect*) OR (TI challenge#) OR (TI issue*) OR (TI barrier#) OR (TI obstacle#) OR (TI problem*) OR (TI involvement) OR (TI access*) OR (TI engagement) OR ((TI cultur*) N3 ((TI competen*) OR (TI safe*) OR (TI appropriate*) OR (TI sensitiv*))) OR (TI communication*) OR (TI language*) OR (TI interpreter#) OR (TI enabler#) OR (TI facilita*) OR (TI promoter#) OR (TI opportunit*) OR (TI advantage#) OR (TI disadvantage#)) |
| S2 | (MH "Patient Satisfaction+") AND TX ((survey# OR questionnaire#)) |
| S1 | (MH "Qualitative Studies+") OR (MH "Focus Groups") OR (MH "Interviews+") OR (MH "Participant Observation") OR (MH "Open-Ended Questionnaires") OR (MH "Meta Synthesis") |

#### Date: 06/10/2025

| S5 | S3 AND S2 AND S1 |
| --- | --- |
| S4 | S3 AND S2 AND S1 |
| S3 | ((MH "Hospitals, Rural") OR (MH "Rural Population") OR (MH "Rural Health") OR (MH "Rural Health Centers") OR (MH "Rural Health Services") OR (MH "Rural Areas") OR (MH "Rural Nursing") OR (MH "Rural Health Personnel") OR (MH "Rural Nurses")) OR (((TI rural* OR AB rural* OR SU rural*) OR (TI regional OR AB regional OR SU regional) OR (TI non-urban OR AB non-urban OR SU non-urban) OR (TI non-metro* OR AB non-metro* OR SU non-metro*) OR (TI coastal OR AB coastal OR SU coastal) OR (TI outport# OR AB outport# OR SU outport#) OR (((TI remote OR AB remote OR SU remote) OR (TI isolated OR AB isolated OR SU isolated)) N3 ((TI area# OR AB area# OR SU area#) OR (TI communit* OR AB communit* OR SU communit*) OR (TI local* OR AB local* OR SU local*) OR (TI population# OR AB population# OR SU population#) OR (TI region# OR AB region# OR SU region#) OR (TI settlement# OR AB settlement# OR SU settlement#) OR (TI setting# OR AB setting# OR SU setting#) OR (TI town# OR AB town# OR SU town#) OR (TI village# OR AB village# OR SU village#))))) |
| S2 | TX ((refugee# OR "asylum seeker#" OR (humanitarian N2 (entrant# OR migrant# OR immigrant#)) OR (displaced N3 (person# OR people OR population#)) OR ((migrant# OR immigrant#) N3 (forced OR cris#s OR disaster* OR war# OR conflict* OR persecut* OR escap* OR flee* OR evacuat* OR political)))) |
| S1 | ((MH "Qualitative Studies+") OR (MH "Focus Groups") OR (MH "Interviews+") OR (MH "Participant Observation") OR (MH "Open-Ended Questionnaires") OR (MH "Meta Synthesis")) OR ((MH "Patient Satisfaction+") AND TX ((survey# OR questionnaire#))) OR (((TI satisfaction#) OR (TI perce*) OR (TI experience#) OR (TI attitude#) OR (TI view*) OR (TI opinion#) OR (TI preference#) OR (TI expect*) OR (TI challenge#) OR (TI issue*) OR (TI barrier#) OR (TI obstacle#) OR (TI problem*) OR (TI involvement) OR (TI access*) OR (TI engagement) OR ((TI cultur*) N3 ((TI competen*) OR (TI safe*) OR (TI appropriate*) OR (TI sensitiv*))) OR (TI communication*) OR (TI language*) OR (TI interpreter#) OR (TI enabler#) OR (TI facilita*) OR (TI promoter#) OR (TI opportunit*) OR (TI advantage#) OR (TI disadvantage#))) OR ((TI qualitative OR SU qualitative) OR ((AB qualitative) N3 ((AB stud*) OR (AB data) OR (AB finding#) OR (AB evaluation) OR (AB analysis))) OR ((TI interview* OR AB interview* OR SU interview*) OR (TI "focus group*" OR AB "focus group*" OR SU "focus group*") OR (((TI unstructured OR AB unstructured OR SU unstructured) OR (TI semi-structured OR AB semi-structured OR SU semi-structured) OR (TI semistructured OR AB semistructured OR SU semistructured) OR (TI open OR AB open OR SU open)) N2 (TI question* OR AB question* OR SU question*)) OR (TI "case stud*" OR AB "case stud*" OR SU "case stud*")) OR ((TI "grounded theory" OR AB "grounded theory" OR SU "grounded theory") OR (TI ethnograph* OR AB ethnograph* OR SU ethnograph*) OR (TI phenomenolog* OR AB phenomenolog* OR SU phenomenolog*) OR (TI ipa OR AB ipa OR SU ipa) OR (TI narratives OR AB narratives OR SU narratives) OR (TI "narrative analysis" OR AB "narrative analysis" OR SU "narrative analysis") OR (TI themes OR AB themes OR SU themes) OR (TI "thematic analysis" OR AB "thematic analysis" OR SU "thematic analysis") OR (TI NVivo OR AB NVivo OR SU NVivo)) OR ((TI "mixed method*" OR AB "mixed method*" OR SU "mixed method*") OR (TI "multi* method*" OR AB "multi* method*" OR SU "multi* method*") OR (TI multimethod* OR AB multimethod* OR SU multimethod*) OR (TI "mixed stud*" OR AB "mixed stud*" OR SU "mixed stud*")) OR (((TI qualitative OR AB qualitative OR SU qualitative) N2 (TI review OR AB review OR SU review)) OR (TI metaethnograph* OR AB metaethnograph* OR SU metaethnograph*) OR (TI meta-ethnograph* OR AB meta-ethnograph* OR SU meta-ethnograph*) OR (TI metasynthes* OR AB metasynthes* OR SU metasynthes*) OR (TI meta-synthes* OR AB meta-synthes* OR SU meta-synthes*))) |

### Search in Embase (OvidSP)

#### Date: 26/06/2024

| 1 | grounded theory/ or exp qualitative research/ |
| --- | --- |
| 2 | focus groups/ or interviews as topic/ |
| 3 | exp patient satisfaction/ and (survey? or questionnaire?).mp. |
| 4 | (satisfaction? or perce* or experience? or attitude? or view* or opinion? or preference? or expect* or challenge? or issue* or barrier? or obstacle? or problem* or involvement or access* or engagement or (cultur* adj3 (competen* or safe* or appropriate* or sensitiv*)) or communication* or language* or interpreter? or enabler? or facilita* or promoter? or opportunit* or advantage? or disadvantage?).ti,kf. |
| 5 | qualitative.ti,kf. or (qualitative adj3 (stud* or data or finding? or evaluation or analysis)).ab. |
| 6 | (interview* or focus group* or ((unstructured or semi-structured or semistructured or open) adj2 question*) or case stud*).ti,ab,kf. |
| 7 | (grounded theory or ethnograph* or phenomenolog* or ipa or narratives or narrative analysis or themes or thematic analysis or NVivo).ti,ab,kf. |
| 8 | (mixed method* or multi* method* or multimethod* or mixed stud*).ti,ab,kf. |
| 9 | ((qualitative adj2 review) or metaethnograph* or meta-ethnograph* or metasynthes* or meta-synthes*).ti,ab,kf. |
| 10 | 1 or 2 or 3 or 4 or 5 or 6 or 7 or 8 or 9 |
| 11 | (refugee? or "asylum seeker?" or (humanitarian adj2 (entrant? or migrant? or immigrant?)) or (displaced adj3 (person? or people or population?)) or ((migrant? or immigrant?) adj3 (forced or cris?s or disaster* or war? or conflict* or persecut* or escap* or flee* or evacuat* or political))).mp. |
| 12 | (rural* or regional or non-urban or non-metro* or coastal or outport? or ((remote or isolated) adj3 (area? or communit* or local* or population? or region? or settlement? or setting? or town? or village?))).ti,ab,kf. |
| 13 | Rural Health/ or exp Rural Health Services/ or Hospital, Rural/ or Rural Population/ |
| 14 | 12 or 13 |
| 15 | 10 and 11 and 14 |
| 16 | limit 15 to english language |

#### Date: 06/10/2025

| 1 | grounded theory/ or multimethod study/ or naturalistic inquiry/ or exp qualitative research/ |
| --- | --- |
| 2 | exp interview/ or participant observation/ or open ended questionnaire/ |
| 3 | (patient preference/ or patient satisfaction/) and (survey? or questionnaire?).mp. |
| 4 | (satisfaction? or perce* or experience? or attitude? or view* or opinion? or preference? or expect* or challenge? or issue* or barrier? or obstacle? or problem* or involvement or access* or engagement or (cultur* adj3 (competen* or safe* or appropriate* or sensitiv*)) or communication* or language* or interpreter? or enabler? or facilita* or promoter? or opportunit* or advantage? or disadvantage?).ti,kf. |
| 5 | qualitative.ti,kf. or (qualitative adj3 (stud* or data or finding? or evaluation or analysis)).ab. |
| 6 | (interview* or focus group* or ((unstructured or semi-structured or semistructured or open) adj2 question*) or case stud*).ti,ab,kf. |
| 7 | (grounded theory or ethnograph* or phenomenolog* or ipa or narratives or narrative analysis or themes or thematic analysis or NVivo).ti,ab,kf. |
| 8 | (mixed method* or multi* method* or multimethod* or mixed stud*).ti,ab,kf. |
| 9 | ((qualitative adj2 review) or metaethnograph* or meta-ethnograph* or metasynthes* or meta-synthes*).ti,ab,kf. |
| 10 | 1 or 2 or 3 or 4 or 5 or 6 or 7 or 8 or 9 |
| 11 | (refugee? or "asylum seeker?" or (humanitarian adj2 (entrant? or migrant? or immigrant?)) or (displaced adj3 (person? or people or population?)) or ((migrant? or immigrant?) adj3 (forced or cris?s or disaster* or war? or conflict* or persecut* or escap* or flee* or evacuat* or political))).mp. |
| 12 | (rural* or regional or non-urban or non-metro* or coastal or outport? or ((remote or isolated) adj3 (area? or communit* or local* or population? or region? or settlement? or setting? or town? or village?))).ti,ab,kf. |
| 13 | Rural Health/ or exp Rural Health Care/ or Rural Hospital/ or Rural Population/ or urban rural difference/ or exp rural area/ |
| 14 | 12 or 13 |
| 15 | 10 and 11 and 14 |
| 16 | limit 15 to english language |
| 17 | (2024* or 2025* or 2026*).dc,dd,yr. |
| 18 | 16 and 17 |

### Search in Medline (OvidSP)

#### Date: 26/06/2024

| 1 | grounded theory/ or multimethod study/ or naturalistic inquiry/ or exp qualitative research/ |
| --- | --- |
| 2 | exp interview/ or participant observation/ or open ended questionnaire/ |
| 3 | (patient preference/ or patient satisfaction/) and (survey? or questionnaire?).mp. |
| 4 | (satisfaction? or perce* or experience? or attitude? or view* or opinion? or preference? or expect* or challenge? or issue* or barrier? or obstacle? or problem* or involvement or access* or engagement or (cultur* adj3 (competen* or safe* or appropriate* or sensitiv*)) or communication* or language* or interpreter? or enabler? or facilita* or promoter? or opportunit* or advantage? or disadvantage?).ti,kf. |
| 5 | qualitative.ti,kf. or (qualitative adj3 (stud* or data or finding? or evaluation or analysis)).ab. |
| 6 | (interview* or focus group* or ((unstructured or semi-structured or semistructured or open) adj2 question*) or case stud*).ti,ab,kf. |
| 7 | (grounded theory or ethnograph* or phenomenolog* or ipa or narratives or narrative analysis or themes or thematic analysis or NVivo).ti,ab,kf. |
| 8 | (mixed method* or multi* method* or multimethod* or mixed stud*).ti,ab,kf. |
| 9 | ((qualitative adj2 review) or metaethnograph* or meta-ethnograph* or metasynthes* or meta-synthes*).ti,ab,kf. |
| 10 | 1 or 2 or 3 or 4 or 5 or 6 or 7 or 8 or 9 |
| 11 | (refugee? or "asylum seeker?" or (humanitarian adj2 (entrant? or migrant? or immigrant?)) or (displaced adj3 (person? or people or population?)) or ((migrant? or immigrant?) adj3 (forced or cris?s or disaster* or war? or conflict* or persecut* or escap* or flee* or evacuat* or political))).mp. |
| 12 | (rural* or regional or non-urban or non-metro* or coastal or outport? or ((remote or isolated) adj3 (area? or communit* or local* or population? or region? or settlement? or setting? or town? or village?))).ti,ab,kf. |
| 13 | Rural Health/ or exp Rural Health Care/ or Rural Hospital/ or Rural Population/ or urban rural difference/ or exp rural area/ |
| 14 | 12 or 13 |
| 15 | 10 and 11 and 14 |
| 16 | limit 15 to english language |

#### Date: 06/10/2025

| 1 | grounded theory/ or exp qualitative research/ |
| --- | --- |
| 2 | focus groups/ or interviews as topic/ |
| 3 | exp patient satisfaction/ and (survey? or questionnaire?).mp. |
| 4 | (satisfaction? or perce* or experience? or attitude? or view* or opinion? or preference? or expect* or challenge? or issue* or barrier? or obstacle? or problem* or involvement or access* or engagement or (cultur* adj3 (competen* or safe* or appropriate* or sensitiv*)) or communication* or language* or interpreter? or enabler? or facilita* or promoter? or opportunit* or advantage? or disadvantage?).ti,kf. |
| 5 | qualitative.ti,kf. or (qualitative adj3 (stud* or data or finding? or evaluation or analysis)).ab. |
| 6 | (interview* or focus group* or ((unstructured or semi-structured or semistructured or open) adj2 question*) or case stud*).ti,ab,kf. |
| 7 | (grounded theory or ethnograph* or phenomenolog* or ipa or narratives or narrative analysis or themes or thematic analysis or NVivo).ti,ab,kf. |
| 8 | (mixed method* or multi* method* or multimethod* or mixed stud*).ti,ab,kf. |
| 9 | ((qualitative adj2 review) or metaethnograph* or meta-ethnograph* or metasynthes* or meta-synthes*).ti,ab,kf. |
| 10 | 1 or 2 or 3 or 4 or 5 or 6 or 7 or 8 or 9 |
| 11 | (refugee? or "asylum seeker?" or (humanitarian adj2 (entrant? or migrant? or immigrant?)) or (displaced adj3 (person? or people or population?)) or ((migrant? or immigrant?) adj3 (forced or cris?s or disaster* or war? or conflict* or persecut* or escap* or flee* or evacuat* or political))).mp. |
| 12 | (rural* or regional or non-urban or non-metro* or coastal or outport? or ((remote or isolated) adj3 (area? or communit* or local* or population? or region? or settlement? or setting? or town? or village?))).ti,ab,kf. |
| 13 | Rural Health/ or exp Rural Health Services/ or Hospital, Rural/ or Rural Population/ |
| 14 | 12 or 13 |
| 15 | 10 and 11 and 14 |
| 16 | limit 15 to english language |
| 17 | (2024* or 2025* or 2026*).ed,ez,yr. |
| 18 | 16 and 17 |

### Search in PsycINFO (OvidSP)

#### Date: 26/06/2024

| 1 | exp qualitative methods/ or mixed methods research/ or qualitative study.md. |
| --- | --- |
| 2 | interviews/ or focus group interview/ or semi-structured interview/ or direct observation/ or participant observation/ or (focus group or interview).md. |
| 3 | metasynthesis.md. |
| 4 | client satisfaction/ and (survey? or questionnaire?).mp. |
| 5 | (satisfaction? or perce* or experience? or attitude? or view* or opinion? or preference? or expect* or challenge? or issue* or barrier? or obstacle? or problem* or involvement or access* or engagement or (cultur* adj3 (competen* or safe* or appropriate* or sensitiv*)) or communication* or language* or interpreter? or enabler? or facilita* or promoter? or opportunit* or advantage? or disadvantage?).ti,id. |
| 6 | qualitative.ti,id. or (qualitative adj3 (stud* or data or finding? or evaluation or analysis)).ab. |
| 7 | (interview* or focus group* or ((unstructured or semi-structured or semistructured or open) adj2 question*) or case stud*).ti,ab,id. |
| 8 | (grounded theory or ethnograph* or phenomenolog* or ipa or narratives or narrative analysis or themes or thematic analysis or NVivo).ti,ab,id. |
| 9 | (mixed method* or multi* method* or multimethod* or mixed stud*).ti,ab,id. |
| 10 | ((qualitative adj2 review) or metaethnograph* or meta-ethnograph* or metasynthes* or meta-synthes*).ti,ab,id. |
| 11 | 1 or 2 or 4 or 5 or 6 or 7 or 8 or 9 or 10 |
| 12 | (refugee? or "asylum seeker?" or (humanitarian adj2 (entrant? or migrant? or immigrant?)) or (displaced adj3 (person? or people or population?)) or ((migrant? or immigrant?) adj3 (forced or cris?s or disaster* or war? or conflict* or persecut* or escap* or flee* or evacuat* or political))).mp. |
| 13 | (rural* or regional or non-urban or non-metro* or coastal or outport? or ((remote or isolated) adj3 (area? or communit* or local* or population? or region? or settlement? or setting? or town? or village?))).ti,ab,id. |
| 14 | rural environments/ or rural health/ |
| 15 | 13 or 14 |
| 16 | 11 and 12 and 15 |
| 17 | limit 16 to english language |

#### Date: 06/10/2025

| 1 | exp qualitative methods/ or mixed methods research/ or qualitative study.md. |
| --- | --- |
| 2 | interviews/ or focus group interview/ or semi-structured interview/ or direct observation/ or participant observation/ or (focus group or interview).md. |
| 3 | metasynthesis.md. |
| 4 | client satisfaction/ and (survey? or questionnaire?).mp. |
| 5 | (satisfaction? or perce* or experience? or attitude? or view* or opinion? or preference? or expect* or challenge? or issue* or barrier? or obstacle? or problem* or involvement or access* or engagement or (cultur* adj3 (competen* or safe* or appropriate* or sensitiv*)) or communication* or language* or interpreter? or enabler? or facilita* or promoter? or opportunit* or advantage? or disadvantage?).ti,id. |
| 6 | qualitative.ti,id. or (qualitative adj3 (stud* or data or finding? or evaluation or analysis)).ab. |
| 7 | (interview* or focus group* or ((unstructured or semi-structured or semistructured or open) adj2 question*) or case stud*).ti,ab,id. |
| 8 | (grounded theory or ethnograph* or phenomenolog* or ipa or narratives or narrative analysis or themes or thematic analysis or NVivo).ti,ab,id. |
| 9 | (mixed method* or multi* method* or multimethod* or mixed stud*).ti,ab,id. |
| 10 | ((qualitative adj2 review) or metaethnograph* or meta-ethnograph* or metasynthes* or meta-synthes*).ti,ab,id. |
| 11 | 1 or 2 or 4 or 5 or 6 or 7 or 8 or 9 or 10 |
| 12 | (refugee? or "asylum seeker?" or (humanitarian adj2 (entrant? or migrant? or immigrant?)) or (displaced adj3 (person? or people or population?)) or ((migrant? or immigrant?) adj3 (forced or cris?s or disaster* or war? or conflict* or persecut* or escap* or flee* or evacuat* or political))).mp. |
| 13 | (rural* or regional or non-urban or non-metro* or coastal or outport? or ((remote or isolated) adj3 (area? or communit* or local* or population? or region? or settlement? or setting? or town? or village?))).ti,ab,id. |
| 14 | rural environments/ or rural health/ |
| 15 | 13 or 14 |
| 16 | 11 and 12 and 15 |
| 17 | limit 16 to english language |
| 18 | (2024* or 2025* or 2026*).up,yr. |
| 19 | 17 and 18 |

### Search in Social Science Citation Index (Web of Science Core Collection)

#### Date: 26/06/2024

| 1 | (TI=(satisfaction$ OR perce* OR experience$ OR attitude$ OR view* OR opinion$ OR preference$ OR expect* OR challenge$ OR issue* OR barrier$ OR obstacle$ OR problem* OR involvement OR access* OR engagement OR (cultur* NEAR/3 (competen* OR safe* OR appropriate* OR sensitiv*)) OR communication* OR language* OR interpreter$ OR enabler$ OR facilita* OR promoter$ OR opportunit* OR advantage$ OR disadvantage$) AND TS=(survey$ OR questionnaire$)) OR TI=qualitative OR AB=(qualitative NEAR/3 (stud* OR data OR finding$ OR evaluation OR analysis)) OR TS=(interview* OR "focus group*" OR ((unstructured OR semi-structured OR semistructured OR open) NEAR/2 question*) OR "case stud*") OR TS=("grounded theory" OR ethnograph* OR phenomenolog* OR ipa OR narratives OR "narrative analysis" OR themes OR "thematic analysis" OR NVivo) OR TS=("mixed method*" OR "multi* method*" OR multimethod* OR "mixed stud*") OR TS=((qualitative NEAR/2 review) OR metaethnograph* OR meta-ethnograph* OR metasynthes* OR meta-synthes*) Editions: WOS.SSCI |
| --- | --- |
| 2 | TS=(refugee$ OR "asylum seeker$" OR (humanitarian NEAR/2 (entrant$ OR migrant$ OR immigrant$)) OR (displaced NEAR/3 (person$ OR people OR population$)) OR ((migrant$ OR immigrant$) NEAR/3 (forced OR cris$s OR disaster* OR war$ OR conflict* OR persecut* OR escap* OR flee* OR evacuat* OR political))) Editions: WOS.SSCI |
| 3 | TS=(rural* OR regional OR non-urban OR non-metro* OR coastal OR outport$ OR ((remote OR isolated) NEAR/3 (area$ OR communit* OR local* OR population$ OR region$ OR settlement$ OR setting$ OR town$ OR village$))) Editions: WOS.SSCI |
| 4 | #3 AND #2 AND #1 Editions: WOS.SSCI |
| 5 | #3 AND #2 AND #1 and English (Languages) Editions: WOS.SSCI |

#### Date: 06/10/2025

| 1 | (TI=(satisfaction$ OR perce* OR experience$ OR attitude$ OR view* OR opinion$ OR preference$ OR expect* OR challenge$ OR issue* OR barrier$ OR obstacle$ OR problem* OR involvement OR access* OR engagement OR (cultur* NEAR/3 (competen* OR safe* OR appropriate* OR sensitiv*)) OR communication* OR language* OR interpreter$ OR enabler$ OR facilita* OR promoter$ OR opportunit* OR advantage$ OR disadvantage$) AND TS=(survey$ OR questionnaire$)) OR TI=qualitative OR AB=(qualitative NEAR/3 (stud* OR data OR finding$ OR evaluation OR analysis)) OR TS=(interview* OR "focus group*" OR ((unstructured OR semi-structured OR semistructured OR open) NEAR/2 question*) OR "case stud*") OR TS=("grounded theory" OR ethnograph* OR phenomenolog* OR ipa OR narratives OR "narrative analysis" OR themes OR "thematic analysis" OR NVivo) OR TS=("mixed method*" OR "multi* method*" OR multimethod* OR "mixed stud*") OR TS=((qualitative NEAR/2 review) OR metaethnograph* OR meta-ethnograph* OR metasynthes* OR meta-synthes*) Editions: WOS.SSCI |
| --- | --- |
| 2 | TS=(refugee$ OR "asylum seeker$" OR (humanitarian NEAR/2 (entrant$ OR migrant$ OR immigrant$)) OR (displaced NEAR/3 (person$ OR people OR population$)) OR ((migrant$ OR immigrant$) NEAR/3 (forced OR cris$s OR disaster* OR war$ OR conflict* OR persecut* OR escap* OR flee* OR evacuat* OR political))) Editions: WOS.SSCI |
| 3 | TS=(rural* OR regional OR non-urban OR non-metro* OR coastal OR outport$ OR ((remote OR isolated) NEAR/3 (area$ OR communit* OR local* OR population$ OR region$ OR settlement$ OR setting$ OR town$ OR village$))) Editions: WOS.SSCI |
| 4 | #3 AND #2 AND #1 Editions: WOS.SSCI |
| 5 | #3 AND #2 AND #1 Editions: WOS.SSCI Timespan: 2024-01-01 to 2025-12-31 |
| 6 | #3 AND #2 AND #1 and English (Languages) Editions: WOS.SSCI Timespan: 2024-01-01 to 2025-12-31 |
