# Supplementary material for: Healthcare experiences of refugees and asylum-seekers resettled in non-metropolitan high-income settings: a systematic review and thematic synthesis of qualitative evidence
Source: Int J Equity Health. 2026 Jul 14;25:238. doi: 10.1186/s12939-026-02933-9 (PMC13651060; doi:10.1186/s12939-026-02933-9)
Supplement: Supplementary file 2 [file 12939_2026_2933_MOESM2_ESM.docx]

## Additional File 2: CASP Checklist

| Article | Item 1: Clear research aim | Item 2: Qualitative methodology appropriate | Item 3: Design appropriate | Item 4: Recruitment strategy appropriate | Item 5:  Data collection appropriate | Item 6: Researcher-participant relationships | Item 7: Ethical issues discussed | Item 8: Data analysis rigorous | Item 9: Clear findings statement | Item 10: Value of this research |
| --- | --- | --- | --- | --- | --- | --- | --- | --- | --- | --- |
| Au et al., 2021 | Yes | Yes | Yes | Yes | Yes | No | Yes | Yes | Yes | Yes |
| Correa-Velez et al., 2013 | Yes | Yes | Yes | Yes | Yes | No | Yes | Yes | Yes | Yes |
| Eid et al., 2025 | Yes | Yes | Yes | Yes | Yes | Yes | Yes | Yes | Yes | Yes |
| Grønseth, 2006 | Yes | Yes | Yes | Yes | Yes | Yes | Yes | Yes | Yes | Yes |
| Hahn et al., 2020 | Yes | Yes | Yes | Yes | Yes | Yes | Yes | Yes | Yes | Yes |
| Hawkes et al., 2021a † | Yes | Yes | Yes | Yes | Yes | No | Yes | Yes | Yes | Yes |
| Hawkes et al., 2021b † | Yes | Yes | Yes | Yes | Yes | No | Yes | Yes | Yes | Yes |
| Kelley et al., 2024 | Yes | Yes | Yes | Can’t tell | Yes | No | Yes | Yes | Yes | Yes |
| Lloyd et al., 2014 | Yes | Yes | Yes | Yes | Yes | No | Yes | Yes | Yes | Yes |
| Mwanri et al., 2023 ‡ | Yes | Yes | Yes | Yes | Yes | No | Yes | Yes | Yes | Yes |
| Shrestha-Ranjit et al., 2020 | Yes | Yes | Yes | Yes | Yes | Yes | Yes | Yes | Yes | Yes |
| Smith et al., 2019 § | Yes | Yes | Yes | Yes | Yes | No | No | Yes | Yes | Yes |
| Smith et al., 2020 § | Yes | Yes | Yes | Yes | Yes | No | Yes | Yes | Yes | Yes |
| Sypek et al., 2008 | Yes | Yes | Yes | Yes | Yes | No | Yes | Yes | Yes | Yes |
| White et al., 2025 | Yes | Yes | Yes | Yes | Yes | Yes | Yes | Yes | Yes | Yes |
| Wood et al., 2019 | Yes | Yes | Yes | Yes | Yes | No | Yes | Yes | Yes | Yes |
| Ziersch et al., 2020 ‡ | Yes | Yes | Yes | Yes | Yes | Yes | Yes | Yes | Yes | Yes |

† articles form part of the same study

‡ articles form part of the same study

§ articles form part of the same study
