# Supplementary material for: Healthcare experiences of refugees and asylum-seekers resettled in non-metropolitan high-income settings: a systematic review and thematic synthesis of qualitative evidence
Source: Int J Equity Health. 2026 Jul 14;25:238. doi: 10.1186/s12939-026-02933-9 (PMC13651060; doi:10.1186/s12939-026-02933-9)
Supplement: Supplementary file 3 [file 12939_2026_2933_MOESM3_ESM.docx]

# Additional File 3: Code List

| **Code** | **Sources** | **References** |
| --- | --- | --- |
| 1. Opportunities of new gender roles | Shrestha-Ranjit et al., 2020  Smith et al., 2020 | 4 |
| 2. Stereotypes, racism and assumptions about refugees | Au et al., 2021  Correa-Velez et al., 2013  Eid et al., 2025  Grønseth, 2006  Hahn et al., 2020  Hawkes et al., 2021a  Mwanri et al., 2023  Smith et al., 2020  Wood et al., 2019  Ziersch et al., 2020 | 35 |
| 3. Stress associated with the changes to social and cultural norms | Au et al., 2021  Correa-Velez et al., 2013  Eid et al., 2025  Hawkes et al., 2021a  Hawkes et al., 2021b  Lloyd et al., 2014  Mwanri et al., 2023  Smith et al., 2020  Wood et al., 2019  Ziersch et al., 2020 | 23 |
| 4. Stress of losing and dishonouring cultural heritage | Grønseth, 2006  Hawkes et al., 2021b  Smith et al., 2020  Wood et al., 2019 | 7 |
| 5. Stress of traditional and changing gender roles | Au et al., 2021  Grønseth, 2006  Hawkes et al., 2021a  Hawkes et al., 2021b  Shrestha-Ranjit et al., 2020  Smith et al., 2020  White et al., 2025  Wood et al., 2019 | 15 |
| 6. Past trauma and its emotional impacts affect daily functioning | Grønseth, 2006  Hawkes et al., 2021a  Smith et al., 2019  Smith et al., 2020  White et al., 2025  Wood et al., 2019 | 11 |
| 7. Stress of family, friends and community left behind | Correa-Velez et al., 2013  Hawkes et al., 2021a  Smith et al., 2019  Smith et al., 2020  White et al., 2025  Wood et al., 2019 | 8 |
| 8. Age and carer-responsibilities as a barrier to language learning | Au et al., 2021  Hawkes et al., 2021b  Smith et al., 2020  White et al., 2025 | 9 |
| 9. Stress from age-based rather than ability-based expectations for educational ability | Smith et al., 2020 | 1 |
| 10. Lack of education as a barrier to English language learning | Hawkes et al., 2021a  Hawkes et al., 2021b  Smith et al., 2020 | 4 |
| 11. Lack of English as barrier and stressor during resettlement | Au et al., 2021  Eid et al., 2025  Hawkes et al., 2021a  Hawkes et al., 2021b  Mwanri et al., 2023  Shrestha-Ranjit et al., 2020  Smith et al., 2020  White et al., 2025  Wood et al., 2019  Ziersch et al., 2020 | 21 |
| 12. Role of English language proficiency to successful resettlement | Eid et al., 2025  Hawkes et al., 2021b  Smith et al., 2020  White et al., 2025  Wood et al., 2019  Ziersch et al., 2020 | 12 |
| 13. Stress and challenges of learning English | Eid et al., 2025  Shrestha-Ranjit et al., 2020  Ziersch et al., 2020 | 3 |
| 14. Appreciation that health services are provided- compared to home countries | Au et al., 2021  Eid et al., 2025  Kelley et al., 2024  Lloyd et al., 2014  Smith et al., 2019  Smith et al., 2020  White et al., 2025  Ziersch et al., 2020 | 25 |
| 15. Challenges of complex health systems and pathways- including long wait times | Au et al., 2021  Correa-Velez et al., 2013  Eid et al., 2025  Hahn et al., 2020  Kelley et al., 2024  Lloyd et al., 2014  Mwanri et al., 2023  White et al., 2025  Ziersch et al., 2020 | 31 |
| 16. Challenges of cultural differences related to healthcare | Au et al., 2021  Eid et al., 2025  Grønseth, 2006  Hawkes et al., 2021a  Lloyd et al., 2014  Shrestha-Ranjit et al., 2020  Smith et al., 2019  Smith et al., 2020  Sypek et al., 2008  Wood et al., 2019  Ziersch et al., 2020 | 28 |
| 17. Collective understandings of health | Kelley et al., 2024  Lloyd et al., 2014 | 13 |
| 18. Cultural support workers and community members filling service gaps - including opportunities for this | Au et al., 2021  Hawkes et al., 2021b  Kelley et al., 2024  Lloyd et al., 2014  Mwanri et al., 2023  Shrestha-Ranjit et al., 2020  White et al., 2025  Ziersch et al., 2020 | 29 |
| 19. Digital and written resources filling health knowledge gaps - especially language gaps | Lloyd et al., 2014 | 6 |
| 20. Experiences of domestic violence | Grønseth, 2006 | 3 |
| 21. Importance of culturally appropriate and trauma-informed healthcare | Au et al., 2021  Hawkes et al., 2021a  Hawkes et al., 2021b  Lloyd et al., 2014  Shrestha-Ranjit et al., 2020  Sypek et al., 2008  White et al., 2025  Ziersch et al., 2020 | 27 |
| 22. Importance of government funding to facilitate healthcare access- including financial barrier to access | Hahn et al., 2020  Kelley et al., 2024  White et al., 2025 | 8 |
| 23. Importance of health and access to care for refugee resettlement | Au et al., 2021  Hawkes et al., 2021b  Kelley et al., 2024  Smith et al., 2020  White et al., 2025 | 10 |
| 24. Insufficient health and social support resources for refugees | Au et al., 2021  Correa-Velez et al., 2013  Hahn et al., 2020  Hawkes et al., 2021b  Kelley et al., 2024  Lloyd et al., 2014  Sypek et al., 2020  Wood et al., 2019  Ziersch et al., 2020 | 17 |
| 25. Integration into host health systems - including combining traditional and western healthcare | Kelley et al., 2024  White et al., 2025 | 11 |
| 26. Language barriers impacting access to health services | Au et al., 2021  Eid et al., 2025  Hawkes et al., 2021b  Kelley et al., 2024  Lloyd et al., 2014  Mwanri et al., 2023  Shrestha-Ranjit et al., 2020  Smith et al., 2020  Sypek et al., 2020  Wood et al., 2019  Ziersch et al., 2020 | 54 |
| 27. Limited health literacy and education as barrier to care | Eid et al., 2025  Kelley et al., 2024  Lloyd et al., 2014  White et al., 2025  Wood et al., 2019 | 11 |
| 28. Mistrust of healthcare providers in host community | Au et al., 2021  Eid et al., 2025  Grønseth, 2006  Lloyd et al., 2014  Sypek et al., 2020 | 9 |
| 29. Negative perceptions of mental health conditions and healthcare | Eid et al., 2025  Grønseth, 2006  Hawkes et al., 2021a | 12 |
| 30. Racism and discrimination in healthcare settings | Au et al., 2021  Hahn et al., 2020 | 4 |
| 31. Refugees falling through health service gaps | Au et al., 2021  Correa-Velez et al., 2013  Hawkes et al., 2021b  Kelley et al., 2024  Lloyd et al., 2014  Mwanri et al., 2023  White et al., 2025  Wood et al., 2019 | 15 |
| 32. Resettlement providers helping refugees access healthcare | Lloyd et al., 2014  White et al., 2025  Ziersch et al., 2020 | 13 |
| 33. Stress and challenge of travel to health services | Hahn et al., 2020  Kelley et al., 2024  Mwanri et al., 2023  Sypek et al., 2020  White et al., 2025  Ziersch et al., 2020 | 14 |
| 34. Benefits of employment and volunteering opportunities | Mwanri et al., 2023  Smith et al., 2020  Wood et al., 2019  Ziersch et al., 2020 | 20 |
| 35. Disempowerment to advocate or change circumstances | Grønseth, 2006  Lloyd et al., 2014  Mwanri et al., 2023  White et al., 2025 | 6 |
| 36. Government role in addressing employment issues | Correa-Velez et al., 2013  Smith et al., 2020  Wood et al., 2019 | 6 |
| 37. Language barriers contributing to non-medical stressors | Hawkes et al., 2021a  Hawkes et al., 2021b  Shrestha-Ranjit et al., 2020  Smith et al., 2020  Wood et al., 2019  Ziersch et al., 2020 | 10 |
| 38. Limitations of regional area causing issues | Au et al., 2021  Correa-Velez et al., 2013  Hahn et al., 2020  Hawkes et al., 2021b  Mwanri et al., 2023  Wood et al., 2019  Ziersch et al., 2020 | 18 |
| 39. Limited transport options and distance in regional areas causing issues | Kelley et al., 2024  White et al., 2025  Ziersch et al., 2020 | 8 |
| 40. Stress and challenges of employment and education- including unemployment | Correa-Velez et al., 2013  Grønseth, 2006  Hawkes et al., 2021b  Mwanri et al., 2023  Shrestha-Ranjit et al., 2020  Smith et al., 2020  Wood et al., 2019  Ziersch et al., 2020 | 48 |
| 41. Stress and challenges of finances | Correa-Velez et al., 2013  Hawkes et al., 2021a  Hawkes et al., 2021b  Kelley et al., 2024  Lloyd et al., 2014  Wood et al., 2019  Ziersch et al., 2020 | 16 |
| 42. Stress and challenges of housing access | Correa-Velez et al., 2013  Hawkes et al., 2021a  Hawkes et al., 2021b  Mwanri et al., 2023  Kelley et al., 2024  Smith et al., 2020  White et al., 2025  Ziersch et al., 2020 | 16 |
| 43. Stress and challenges of not-recognising overseas qualifications and education | Mwanri et al., 2023  Shrestha-Ranjit et al., 2020  Wood et al., 2019  Ziersch et al., 2020 | 10 |
| 44. Positive emotions associated with new home | Correa-Velez et al., 2013  Eid et al., 2025  Smith et al., 2019  Smith et al., 2020  White et al., 2025  Wood et al., 2019  Ziersch et al., 2020 | 18 |
| 45. Positive impacts of a small regional area | Au et al., 2021  Mwanri et al., 2023 | 6 |
| 46. Benefits of cultural stereotyping and community reputation by host communities | Mwanri et al., 2023  Ziersch et al., 2020 | 5 |
| 47. Importance of connection to and support from local host community | Correa-Velez et al., 2013  Eid et al., 2025  Hawkes et al., 2021a  Hawkes et al., 2021b  Lloyd et al., 2014  Mwanri et al., 2023  Smith et al., 2020  White et al., 2025  Wood et al., 2019  Ziersch et al., 2020 | 30 |
| 48. Importance of connection to faith-based groups | Eid et al., 2025  Lloyd et al., 2014  Mwanri et al., 2023  Ziersch et al., 2020 | 9 |
| 49. Importance of connection to settlement service providers | Eid et al., 2025  Hawkes et al., 2021b  Lloyd et al., 2014  Mwanri et al., 2023  White et al., 2025  Wood et al., 2019  Ziersch et al., 2020 | 19 |
| 50. Importance of connection to their refugee community | Au et al., 2021  Eid et al., 2025  Kelley et al., 2024  Lloyd et al., 2014  Mwanri et al., 2023  Smith et al., 2020  White et al., 2025  Wood et al., 2019  Ziersch et al., 2020 | 43 |
| 51. Stress and challenge of disconnection and loneliness during resettlement | Correa-Velez et al., 2013  Grønseth, 2006  Hawkes et al., 2021a  Lloyd et al., 2014  Mwanri et al., 2023  White et al., 2025  Ziersch et al., 2020 | 16 |
