# Supplementary material for: Healthcare experiences of refugees and asylum-seekers resettled in non-metropolitan high-income settings: a systematic review and thematic synthesis of qualitative evidence
Source: Int J Equity Health. 2026 Jul 14;25:238. doi: 10.1186/s12939-026-02933-9 (PMC13651060; doi:10.1186/s12939-026-02933-9)
Supplement: Supplementary file 4 [file 12939_2026_2933_MOESM4_ESM.docx]

## Additional File 4: Descriptive and Analytical Themes

| Descriptive theme | Explanation |
| --- | --- |
| Cultural and personal identity shifts after resettlement | Refugees encounter stress balancing cultural heritage with adapting to new norms, often encountering racism, stereotypes, and shifting gender roles. While new opportunities arise (e.g. women accessing work and education), they are complicated by identity loss and distrust from host communities.  **Codes:**  1. Opportunities for new gender roles  2. Stereotypes, racism, and assumptions about refugees  3. Stress associated with changes to social and cultural norms  4. Stress of losing and dishonouring cultural heritage  5. Stress of traditional and changing general roles  **Corresponding Analytical Theme/s:**   - **Power of the Social** - **Domains of Influence** |
| Displacement affects emotional and psychological well-being | Past trauma, separation from loved ones, and isolation impact refugees' mental health. Stigma and disempowerment discourage help-seeking, and somatisation complicates care, especially in rural areas where mental health services are often scarce.  **Codes:**  6. Past trauma and its emotional impacts affect daily functioning  7. Stress of family, friends and community left behind  29. Negative perceptions of mental health conditions and healthcare  35. Disempowerment to advocate or change circumstances  51. Stress and challenge of disconnection and loneliness during resettlement  **Corresponding Analytical Theme/s:**   - **Power of the Social** - **The Rural Effect** |
| Language remains a major obstacle | Limited English proficiency restricts access to healthcare, education, and employment. Learning English is stressful, especially for those with caregiving responsibilities or little formal education, and older refugees may struggle with language barriers long after resettlement.  **Codes:**  8. Age and carer-responsibilities as a barrier to language learning  10. Lack of education as a barrier to English language learning  11. Lack of English as a barrier and stressor during resettlement  12. Role of English language proficiency to successful resettlement  13. Stress and challenges of learning English  26. Language barriers impacting access to health services  37. Language barriers contributing to non-medical stressors  **Corresponding Analytical Theme/s:**   - **Domains of Influence** |
| Navigating the health system is complex | Refugees appreciate that healthcare is available but struggle with complex systems, long wait times, and limited services. Mistrust and resource shortages create barriers, though co-located services in rural and regional areas can improve access. Over time, as they learn the new systems refugees integrate traditional and Western health practices.  **Codes:**  14. Appreciation that health services are provided compared to home countries  15. Challenges of complex health systems and pathways, including long wait times  22. Importance of government funding to facilitate healthcare access, including financial barriers  24. Insufficient health and social support resources for refugees  25. Integration into host health systems, including combining traditional and western healthcare  31. Refugees falling through health service gaps  **Corresponding Analytical Theme/s:**   - **Domains of Influence** - **The Rural Effect** |
| Care in health settings must be safe, accessible, and culturally appropriate | Healthcare quality significantly affects refugee experiences. Culturally appropriate, trauma-informed care is often lacking, whilst language barriers and health literacy gaps make care provision difficult. Racism within healthcare settings further erodes trust, compounding the challenge of limited resourcing and leaving community and social networks to fill critical gaps.  **Codes:**  16. Challenges of cultural differences related to healthcare  17. Collective understandings of health  18. Cultural support workers and community members filling service gaps, including opportunities for this  21. Importance of culturally appropriate and trauma-informed healthcare  26. Language barriers impacting access to health services  27. Limited health literacy and education as a barrier to care  28. Mistrust of healthcare providers in host community  30. Racism and discrimination in healthcare settings  32. Resettlement providers helping refugees access healthcare  **Corresponding Analytical Theme/s:**   - **Domains of Influence** - **Power of the Social** |
| Employment is key to financial security and healthcare access | Employment is key to financial security and healthcare access, yet refugees face employment challenges like qualification non-recognition, unemployment, and financial stress. Volunteering helps build skills but risks exploitation, while age-based education placement hinders career prospects.  **Codes:**  9. Stress from age-based rather than ability-based expectations for educational ability  34. Benefits of employment and volunteering opportunities  36. Government role in addressing employment issues  40. Stress and challenges of unemployment and education- including unemployment  41. Stress and challenges of finances  43. Stress and challenges of not recognising overseas qualifications and education  **Corresponding Analytical Theme/s:**   - **Domains of Influence** - **The Rural Effect** |
| Housing and transport challenges shape health and access to care | Housing shortages, affordability issues, and vast geographic distance impact refugees’ health, wellbeing, and access to care. Limited public transport in rural and regional areas further restricts access. Cultural stereotypes influence housing access, acting as either an enabler or barrier depending on community stereotypes.  **Codes:**  2. Stereotypes, racism and assumptions about refugees  3. Stress and challenge of travelling to health services  4. Limitations of regional areas causing issues  39. Limited transport options in regional areas causing difficulties  42. Stress and challenges of housing access  46. Benefits of cultural stereotyping and community reputation by host communities  **Corresponding Analytical Theme/s:**   - **The Rural Effect** - **Domains of Influence** |
